# Characterisation of seminal plasma-derived mediators involved in PMN evasion of ram spermatozoa *in vitro*

**DOI:** 10.3389/fimmu.2026.1891264

**Published:** 2026-09-03

**Authors:** Sophie Warr, Lydia Mikhael, Elizabeth Torres-Arce, John Schjenken, Taylor Pini, Simon P. de Graaf, Jessica P. Rickard

**Affiliations:** 1 School of Life and Environmental Sciences, Faculty of Science, The University of Sydney, Sydney, NSW, Australia; 2 Centre for Reproductive Science, School of Science, College of Engineering, Science and Environment, University of Newcastle, University Drive, Callaghan, NSW, Australia; 3 Reproductive and Family Health Research Program, Hunter Medical Research Institute, New Lambton Heights, NSW, Australia; 4 School of Veterinary Science, Faculty of Science, The University of Queensland, Gatton, QLD, Australia

**Keywords:** cryopreservation, extracellular vesicle, fertility, immune, sheep

## Abstract

Seminal plasma (SP) is a complex immune modulator within the female reproductive tract of several species. In sheep, it can restore the fertility of cryopreserved sperm and is necessary for the fertility of epididymal sperm following cervical artificial insemination. We have also shown it capable of protecting cryopreserved sperm from polymorphonuclear leukocyte (PMN) binding *in vitro*. Thus, we hypothesize SP, and its components confer immune protection to sperm following deposition in the ovine cervix and that sperm cryopreservation disrupts this delicate sperm-female interaction. To delineate the contributions of soluble and extracellular vesicle (EV) associated SP components, frozen-thawed ram spermatozoa were incubated with whole SP, EV-depleted SP (DSP) and enriched EVs. All SP treatments significantly reduced binding (FTSP: 20.7 ± 3.39%; FTDSP: 24.3 ± 3.64%; FTEV: 19.8 ± 3.63%) compared to the control (36.3 ± 3.63%; p < 0.0001), with no differences between treated groups. To further isolate inhibitory factors, FT was incubated with SP fractionated into 5 molecular weight treatments (F1^-5^; <10, 10–30, 30–50, 50–100, >100 kDa). Two fractions, F3 (30^-5^0kDa) and F5 (>100kDa) reduced PMN binding (22.44 ± 2.18%, 21.67 ± 1.85%, respectively) compared to the control (FT: 58.83 ± 4.54%; p < 0.0001) and similar to whole SP (20.67 ± 1.65%; p>0.05), indicating these fractions contain key immunoregulatory factors. Proteomic analysis identified 2,161 proteins in whole SP, with SP-EVs contributing 795 unique proteins. Among protective fractions (SP, DSP, EV, F3, F5), 588 proteins were shared, including a core signature of PITPNB, RPLP2, SLC9A3R1, PTTG1IP, CKAP4, and FKBP11, absent in non-protective fractions. Complement regulators (CD46, CD59, CFH, CLU) and glycocalyx-associated proteins (CRISPs, DEFB124) were enriched in protective fractions, supporting layered immunomodulation via both soluble and vesicular delivery. Functional analyses demonstrated that SP modulates PMN binding and activation while maintaining sperm viability, highlighting a structured, multi-layered immunoregulatory system. These findings provide the most comprehensive characterization of ram SP and SP-EVs to date, integrating functional assays with proteomic profiling, and reveal conserved protein signatures that underpin sperm immune tolerance. This work establishes a foundation for future studies targeting EV-mediated delivery and glycocalyx-associated immunoregulators to improve fertility outcomes and assisted reproductive technologies.

## Introduction

Reproductive success relies on the ability of spermatozoa to traverse the immunologically complex female reproductive tract (FRT), negotiating anatomical barriers, mucosal secretions, and active immune surveillance (1). During natural mating, sperm are remarkably equipped to meet this challenge: the female immune system balances effective pathogen defense with permissiveness toward paternal gametes, creating a transient state of immune tolerance. How this selective quiescence is achieved remains poorly understood, representing a critical knowledge gap in early male-female reproductive interactions and our conceptualization of the ‘fertile sperm.’

The fragility of this mechanism is perhaps best highlighted in the sheep, where cervical artificial insemination (AI) with frozen-thawed (FT) ram spermatozoa results in fertility rates of < 30%, compared to >75% with fresh semen (2). This discrepancy is attributed primarily to impaired sperm transit through the cervix, rather than overt deficits in classical sperm parameters such as motility, or viability (3–5). Cryopreservation induces phenotypic alterations that extend beyond these conventional metrics, including modification of sperm membrane-associated proteins (6), which leads to the exposure of paternal antigens (7) on the sperm surface. Additionally, it contributes to dilution or removal of seminal plasma (SP), a fluid known to influence sperm transport and survival (8, 9). Seminal plasma-naïve epididymal sperm also fail to traverse the ovine cervix, further supporting a role for SP in mediating fertility (9). Collectively, cryopreservation and loss of SP produce a sperm phenotype that is molecularly and immunogenically distinct from fresh, seminal plasma exposed ‘fertile’ sperm, leading to a potential failure in permissive mechanisms and increased clearance by the female immune system.

Across mammalian species, semen deposition triggers a rapid and highly conserved inflammatory response in the female reproductive tract, characterized by epithelial receptor activation (10), cytokine and chemokine release (11), fluid influx (12), and leukocyte recruitment (13–15), that together regulate microbial defense, sperm clearance, and reproductive tract conditioning for conception (16). Central to this response is the rapid influx of polymorphonuclear neutrophils (PMNs), which migrate into the cervical and uterine lumen within minutes of semen exposure and interact directly with spermatozoa, a phenomenon documented across multiple species including women (17), mice (18), and a wide range of livestock species, including cows (19), sheep (20), pigs (21) and horses (22). PMN influences early sperm clearance in the mucosa and lumen, where they deploy a combination of phagocytosis, degranulation, and neutrophil extracellular trap (NET) formation to remove spermatozoa and pathogens (20, 23, 24). Complement activation, inflammatory cytokine release, and chemokine-mediated recruitment further coordinate this response (25–27). While PMNs are essential for immune protection, their potent effector functions can compromise sperm survival. *In vitro* studies across bovine (28, 29), equine (30, 31), porcine (32, 33), donkey (34), and human (35) species have documented sperm-PMN interactions, showing sperm elicit potent NET and phagocytic responses, also highlighting the significance of female-derived serum in this function (36). Studies in sheep have been limited (37, 38). Despite robust physiological investigation, the molecular determinants of selective sperm clearance, particularly how sperm evade immune recognition, remain poorly defined across human and livestock models.

Across these studies, seminal plasma has emerged as a key regulator of this early immune dialogue, suppressing sperm-PMN binding and modulating neutrophil activity (36). Studies in domestic species have demonstrated that SP can suppress neutrophil activation, reduce NET formation, and protect viable spermatozoa through antioxidant activity and immunoregulatory proteins (33, 39–41). These findings suggest that SP components function to fine-tune post-mating inflammation, balancing antimicrobial defense with the preservation of fertilization-competent sperm. In the ram the responsible SP protective factors appear to be heat-labile, indicating a pivotal role for proteins (38). SP is highly complex, containing in excess of 700 proteins, lipids, glycans, and extracellular nucleic acids, capable of modulating sperm function and the female immune environment (42–44). Within SP, proteins exist as soluble factors or are packaged within extracellular vesicles (EVs); lipid-bound nanostructures (30–200 nm) that carry proteins, lipids, and nucleic acids and provide a protected mode of delivery to transcriptionally silent sperm or FRT cells (45, 46). EVs are increasingly recognized as modulators of reproductive immunity, capable of delivering bioactive molecules that modify immune environments and support sperm survival (reviewed by (46)). Seminal EVs, including prostasomes and epididymosomes, have been reported to suppress inflammatory responses by reducing reactive oxygen species (ROS) production, modulating cytokine signaling, and transferring immunoregulatory cargo to leukocytes, including PMNs (reviewed by (47, 48)). Through these mechanisms, EVs are proposed to limit excessive PMN activation and sperm binding while preserving antimicrobial defense. However, the contribution of EV-associated factors to SP-mediated sperm protection, particularly in the ram, remains largely unexplored.

Despite clear evidence that SP protects spermatozoa from PMN attack, the specific molecular factors, their mode of delivery (soluble versus EV-associated), and their functional characteristics are unknown. Moreover, the interplay between SP proteins and the innate immune system, including PMN responses, complement regulation, and inflammatory signaling, remains undefined. Identifying these factors has direct relevance for understanding key mechanism of sperm immune tolerance within the female tract and how this could be manipulated to direct strategies to improve the fertility of cryopreserved sperm following cervical AI in sheep and more widely enhancing assisted reproductive technologies (ARTs) for livestock and wildlife.

The present study builds on previous findings demonstrating SP-mediated protection of frozen-thawed (FT) ram sperm against PMN binding *in vitro* (38). Here, we aim to isolate and characterize the specific SP components responsible for this protective effect, examining first the role of soluble proteins or EV-associated proteins, then attempting to correlate individual molecular weight-defined fractions to PMN protection. By combining PMN binding assays with proteomic analyses, this work seeks to define the molecular mechanisms through which SP restores permissive sperm immunity, providing insight into the molecular dialogue between semen and the female reproductive tract and identifying targets to improve post-thaw sperm survival and fertility outcomes.

## Materials and methods

### Experimental design

Two complementary studies were designed to identify mediators in seminal plasma that could be responsible for the protection of frozen–thawed ram spermatozoa from PMN binding. Study 1 compared the protective effect of whole seminal plasma (SP) to its soluble, EV-depleted (DSP) and extracellular vesicle (EV) fractions. Building on these outcomes, Study 2 resolved SP into unique molecular-weight fractions (<10 kDa, 10–30 kDa, 30–50 kDa, 50–100 kDa, and >100 kDa) to localise functional activity and refine the search for candidate mediators. To qualitatively assess the protein components underpinning these effects and integrate findings across both studies, all treatments were analyzed by mass spectrometry using both data-dependent acquisition (DDA) and data-independent acquisition (DIA), enabling presence/absence assessment alongside broader proteomic profiling. Extracellular vesicles were characterised in accordance with MISEV2023 guidelines (49).

### Chemicals

All chemicals were purchased from Sigma-Aldrich (Melbourne, Australia) unless otherwise stated.

### Animal use and ethical approval

Merino ewes (*n* = 2) used for blood collection were kept on a chaff-based diet supplemented with lupins in an animal house at the University of Sydney, Camperdown, NSW, Australia. Mature Merino rams used for seminal plasma collection and preparation (*n* = 24) were kept on pasture at the Sheep Unit, University of Sydney, Camden Campus, Cobbitty. All work was approved by the University of Sydney Animal Ethics Committee (Project No: 2023/2277 and 2023/AE002277).

### Seminal plasma collection and preparation

Seminal plasma was obtained from ejaculates (*n* = 9 per ram) collected from mature Merino rams (*n =* 24) in the presence of a teaser ewe during the breeding season 2024/25. The average ejaculate volume across all rams was 1.25 ± 0.44mL, with a wave motion of 4.65 ± 0.33 across all collections. Accompanying ejaculate characteristics per ram, performed per ejaculate prior to inclusion in the pool can be found in **Supplementary Table 1**.

Following initial subjective assessment of mass motility, samples scoring below 3 (scale 1–5) were excluded prior to clarification. Each ejaculate was processed immediately following collection and centrifuged at 12,000 × g, 4 °C for 10 min. The supernatant was aspirated and centrifuged again at 12,000 × g, 4 °C for a further 10 min to remove any remaining spermatozoa or cellular debris. Based on ejaculate characteristics (**Supplementary Table 1**) and final seminal plasma volume, equal volumes of seminal plasma from rams with equivalent profiles were pooled to generate a single composite seminal plasma sample for downstream analyses. This pooled sample was aliquoted and stored at −80 °C until use. For each experiment, a single aliquot was thawed on ice immediately prior to use.

### Isolation of Ram seminal plasma extracellular vesicles (EVS)

An aliquot of the seminal plasma pool was sequentially centrifuged with increasing velocity at 4 °C to eliminate contaminating cells and cellular debris (500 x g, 5 min; 2,000 x g, 5 min; 2,000 x g, 5 min; 8,000 x g, 5 min; 17,000 x g, 20 min; and repeat centrifugations at 17,000 x g for 10 min until a pellet was no longer visible). Extracellular vesicles were then enriched as previously described by (44, 45). Samples were centrifuged at 100, 000 x g for 90 min in a fixed rotor (Hi-tachi CP100NX Ultracentrifuge). Following ultracentrifugation, the non-pelleted supernatant fraction was collected as extracellular vesicle–depleted seminal plasma (DSP). The pellet was washed with 12 ml of phosphate-buffered saline (PBS; 137 mM NaCl, 3 mM KCl, 8 mM Na2HPO4, 1 mM KH2PO4, pH 7.4, mOsm 295+/^-5^) and centrifuged again at 100,000 x g for 90 min. The resulting pellet was resuspended in 1 ml of PBS, and layered onto a discontinuous density gradient modified from previous studies (comprising 50%, 25%, 12.5%, and 6.25%) OptiPrep (Sigma Aldrich, Melbourne, Australia) suspension diluted in a solution of 0.25 M sucrose, 10 mM Tris HCl, pH 7.5) and centrifuged at 108,300 x g for 16 h at 4 °C. After centrifugation, 12 equivalent fractions (1mL per fraction) were collected. A visually enriched EV band corresponding to fractions 9 and 10, was pooled and retained for downstream analyses. Pooled fractions were washed in PBS by ultracentrifugation (100,000 × g, 120 min). Pellets were solubilized in PBS as required for downstream application.

### Validation of EV characteristics

Nanoparticle tracking analysis was used to determine particle size distribution and concentration of seminal plasma-derived EV samples, as previously described (50). Briefly, EV suspensions were diluted 1:10,000 in 0.1 µM filtered PBS before introduction into the chamber of a ZetaView x30 (Particle Matrix. Meerbusch, Germany). Settings were kept constant between samples (488 nm laser; sensitivity 80; shutter 100), capturing 11 × 60-second videos per sample. Particle size distribution and concentration were determined using ZetaView (version 8.05.16 SP3).

For transmission electron microscopy, purified EV suspensions were fixed in 4% paraformaldehyde (1:1 v/v; 15 min on ice), and 15 µL was applied onto carbon-coated copper grids (ProSciTech, Kirwan, QLD, Australia) and incubated at room temperature for 1 hour. Grids were rinsed three times in 0.1 µm-filtered PBS and post-fixed in 1% glutaraldehyde for 5 min at room temperature, followed by three washes in ultrapure water. Grids were stained with methyl cellulose–uranyl acetate for structural contrast (10 min at RT), washed and air-dried overnight. Samples were imaged using a JEOL JEM-1200EXII transmission electron microscope (JEOL Ltd, Tokyo, Japan) at 50–250 kV.

### Fractionation of seminal plasma

SP fractionation was adapted from (51). Briefly, an aliquot of the seminal plasma pool was sequentially centrifuged with increasing velocity at 4 °C to eliminate contaminating cells and cellular debris (500 x g, 5 min; 2,000 x g, 5 min; 2,000 x g, 5 min; 8000 x g, 5 min; 17,000 x g, 20 min; and repeat centrifugations at 17,000 x g for 10 min until a pellet was no longer visible). The sample was then fractionated based on molecular weight using Amicon Ultra centrifugal filters (Merck, Darmstadt, Germany), following the manufacturer’s instructions. Briefly, samples were centrifuged at 4,000 × g for 10 minutes at 20 °C through a filter with a 10 kDa molecular weight cutoff (MWCO) to collect the <10 kDa fraction. The supernatant was sequentially passed through filters with increasing MWCOs (30 kDa, 50 kDa, 100 kDa), with centrifugation repeated at 4,000 × g for 10 minutes at each step. This process yielded 5 distinct MW fractions: <10 kDa [F1], 10–30 kDa [F2], 30–50 kDa [F3], 50–100 kDa [F4], and >100 kDa [F5].

### Sodium dodecyl sulphate-polyacrylamide gel electrophoresis (SDS-page), silver staining, and immunoblotting

Proteins from SP, DSP and EV samples (Study 1), and from each MW-fractionated SP treatment; F1^-5^ (Study 2), were solubilized in SDS-PAGE extraction buffer (10% SDS, 0.3 M sucrose, 0.375 M Tris-HCl, pH 6.8) supplemented with protease inhibitors (Roche, Basel, Switzerland) and heated at 100 °C for 5 min. Insoluble debris was removed by centrifugation (21,000 × g, 10 min, 4 °C), and protein concentration was determined using the DC Protein Assay (Bio-Rad Laboratories, Hercules, CA). To assess the profile of proteins present in each of the samples, equivalent volumes of each sample was diluted in SDS-PAGE loading buffer (10% w/v SDS, 30% v/v glycerol, 0.35 M Tris-HCl pH 6.8, 0.012% w/v bromophenol blue, 2% v/v β-mercaptoethanol) and 5–10 µg protein per lane was resolved on 4–20% Mini-PROTEAN TGX gels (Bio-Rad Laboratories) at 150 V for 45–50 min.

Following electrophoresis, gels were either silver-stained or transferred to nitrocellulose. For silver staining, gels were fixed (10% methanol, 5% acetic acid), sensitized in sodium thiosulphate, stained with silver nitrate, and developed in sodium carbonate/formaldehyde until bands appeared, before stopping in 1% acetic acid. Gels were rinsed in water and imaged using an ImageQuant LAS-4000 Biomolecular Imager (GE Healthcare, Chicago, IL).

For immunoblotting, gels were transferred to nitrocellulose at 350 mA for 1 h. Membranes were blocked in 5% BSA/TBST (0.1% Tween-20), incubated overnight at 4 °C with anti-flotillin-1 (1:1000 mouse mAb; Merck, Darmstadt, Germany), washed, then incubated with HRP-conjugated goat anti-rabbit IgG (1:2500; Merck, Darmstadt, Germany) for 1 h at room temperature. Following final washes, chemiluminescent signals were developed (ECL Plus; Thermo-Fisher Scientific) and captured using an ImageQuant LAS-4000 system (GE Healthcare).

### Functional sperm-PMN binding assay

#### Isolation of polymorphonuclear lymphocytes from peripheral blood

Blood was collected from the jugular vein of mature Merino ewes (n = 2) into ethylenediamine tetraacetic acid (EDTA)-coated vacutainers (Becton Dickinson, Macquarie Park, New South Wales, Australia) and pooled. Polymorphonuclear neutrophils (PMNs) were isolated as per the protocol outlined by (6) immediately following blood collection. Briefly, blood was layered onto a two-phase gradient of Histopaque-1119 and Histopaque-1077 and centrifuged (3 mL Histopaque-1119, 3 mL Histopaque-1077, 6 mL whole blood, 1,200 x g, 30 min, RT). The PMN layer was recovered and red blood cells removed by 30 s hypotonic lysis with ultra-pure water. PMNs were washed twice in phosphate-buffered saline (PBS; 137 mM NaCl, 3 mM KCl, 8 mM Na2HPO4, 1 mM KH2PO4). Cell viability was determined by Trypan blue exclusion as previously described by (6) and was consistently high (> 95%). Immediately prior to mixing with spermatozoa, PMNs were resuspended to 5 x 10^6^ PMN/mL with PBS only (no serum) or PBS + 15% (v/v) ewe serum (S) for studies one and two.

#### Processing and assessment of frozen-thawed ram spermatozoa

Semen was collected via artificial vagina in the presence of a teaser ewe and assessed immediately for quality by scoring wave motion, concentration, and volume (n = 3 rams × 3 ejaculates). Samples with a wave motion < 3 were excluded. Samples were diluted to 400 x 10^6^ spermatozoa/mL in Salamon’s cryodiluent (300 mM Tris, 28 mM glucose, 104 mM citric acid, 15% (v/v) egg yolk, 5% (v/v) glycerol, pH 7.3, mOsm 295+/^-5^) (52), chilled to 4 °C over 2h, then frozen on dry ice in pellets of 250 µL and stored in liquid nitrogen (52). Pellets were thawed in a 37 °C water bath with agitation for 2 min. Frozen-thawed spermatozoa was then washed using a Percoll gradient (4 mL 35%, 2 mL 70%,; 5 min 200 x g, 15 min 1,000 x g) and the subsequent pellets washed (10 min 800 x g) in tris-citrate-fructose (SSF; 300 mM Tris, 94.7 mM citric acid, 27.8 mM fructose, 1% v/v penicillin and streptomycin) (52).

In study 1, frozen-thawed samples were washed as described above and diluted to 100 x 10^6^ spermatozoa/mL in SSF + 0.3% BSA. A 1:1 (v/v) dilution was then performed with either SSF + 0.3% BSA (FT), seminal plasma (50% v/v FTSP), EV depleted SP (50% v/v FTDSP) or EVs (50% v/v FTEV) with a final sperm concentration of 50 x 10^6^ spermatozoa/mL.

In study 2, frozen-thawed samples were washed as described above and diluted to 100 x 10^6^ spermatozoa/mL in SSF + 0.3% BSA. A 1:1 (v/v) dilution was then performed with either SSF + 0.3% BSA (FT), seminal plasma (50% v/v FTSP) or one of the five SP fractions (50% v/v; 10 kDa, 10–30 kDa, 30–50 kDa, 50–100 kDa, and >100 kDa) with a final sperm concentration of 50 x 10^6^ spermatozoa/mL.

#### Assessment of sperm motility and viability

In both studies, immediately prior to incubation with PMNs, sperm motility was measured using computer-assisted sperm analysis (HT CASA IVOS II (Animal Breeder) Version 1.13.7; Hamilton-Thorne, Beverly, MA) using the appropriate settings for ram spermatozoa (this includes among others; head size 10–42 µm^2^, progressive motility thresholds of straightness 80% and average path velocity 75 μm/s). Samples were further diluted to a concentration of 25 × 10^6^ sperm/mL with PBS + 0.3% BSA before 6 µL was placed on slides warmed to 37 °C (Cell Vu; Millennium Sciences, Mulgrave, Victoria, Australia) and enclosed with a 22×22 mm coverslip. For each sample, eight fields of video recordings were recorded, capturing a minimum of 200 cells (frame rate 60 Hz). Motility and kinematic parameters were subsequently calculated, including total and progressive motility.

Samples were assessed for viability and acrosome integrity at a final concentration of 10×10^6^ sperm/mL using the CytoFLEX flow cytometer (CytExport 2.0 Software, Beckman Coulter; USA), with three lasers: 50 mW 488 nm, 50 nW 638 nm, and 80 mW 405 nm. All samples were stained with the DNA probe Hoechst 33,342 (final concentration 1 µg/mL), detected via a 450/45 BP filter, to gate any possible debris in the sample. Sample preparation for sperm viability and acrosome integrity was performed as previously described (53), by staining a combination of propidium iodide (PI, final concentration 6µM) and fluorescein isothiocyanate peanut agglutinin (FITC-PNA, final concentration 0.4 µg/mL) for 10 min at 37 °C. PI and FITC-PNA fluorescence detection was on 690/50 and 525/40 nm BP filters, respectively. Cells were considered viable with intact acrosomes if they were both PI and FITC-PNA negative (**Supplementary Figure 1**).

#### Incubation of PMNs and sperm treatments

Sperm-PMN assays were conducted as reported by (37), for both studies 1 and 2. Spermatozoa treatments at a concentration of 50 x 10^6^ spermatozoa/mL were further diluted 1:1 (v/v) with isolated PMNs (5 x 10^6^ PMN/mL) in PBS (NS; no serum) or serum (S; 15% v/v), resulting in final concentrations of 25 x 10^6^ spermatozoa/mL, 2.5 x 10^6^ PMN/mL and 7.5% (v/v) serum, 25% seminal plasma treatment (Study 1; SSF, SP, DSP, EV; Study 2; SSF, SP, F1^-5^) respectively. Samples were then incubated in a dry bath incubator (Major Science, Saratoga, CA) at 37 °C. After 60 and 120 min, samples were mixed vigorously with a pipette to dissociate large cell clumps, and a 10 µL aliquot was smeared on a labeled microscope slide and air dried thoroughly.

Air-dried slides were stained with a modified Wright’s stain according to the manufacturer’s instructions (1 min flood, wash twice with deionized water) (37). Slides were examined using a phase-contrast microscope (Olympus CX41, Tokyo, Japan) at 200 x magnification. A total of 200 PMNs were evaluated on each slide and classified as inactive (nucleus intact), active (disrupted nucleus/membrane), and free or bound to ≥1 spermatozoon as described in (38). Data are presented as the percentage of total cells activated (active/200 × 100) ± SEM% and the percentage of total cells bound (bound/200 × 100) ± SEM%.

#### Assessment of PMN viability

In both studies, an aliquot of PMNs were co-incubated with all treatments 1:1 v/v, incubated in a dry bath incubator (Major Science) at 37 °C for 120 minutes, then incubated 1:1 v/v with Trypan blue (5 minutes; room temperature), cell viability was determined on a hemocytometer, counting 100 cells, as previously described (37).

### Mass spectrometry

#### Digestion and preparation of samples for mass spectrometry

For each sample, 20 µg of protein was extracted and solubilised in 1% sodium deoxycholate (SDC), 10mM Tris-2-carboxyethyl-phosphine (TCEP), 40 mM Chloroacetamide (CAA), and 100 mM Tris-HCl (pH 8.5). Samples were heated at 95 °C for 10 min with shaking at 1,000 RPM in a ThermoMixer (Eppendorf, Germany) to ensure complete denaturation and reduction. Following cooling, trypsin (1:50 enzyme-to-protein ratio; 0.4 µg per sample) was added, and digestion was carried for 16 hours at 37 °C with continuous agitation at 850 RPM. Digested samples were held at 4 °C until desalting.

Peptides were subjected to SDB-RPS StageTip desalting (Sigma-Aldrich, 66886-U). StageTips were conditioned sequentially with 100 µL 100% acetonitrile (ACN), 100 µL 30% methanol/1% Trifluoroacetic acid (TFA), and 100 µL 0.2% TFA, centrifuged at 1,000 × g for 1 min per step. Samples were loaded and centrifuged at 1,000 × g for 1 min. Peptides were washed twice with 100 µL 99% ethyl acetate/1% TFA, followed by a final wash with 100 µL 0.2% TFA. Elution was performed with 100 µL 80% ACN/5% ammonium hydroxide, centrifuged at 1,000 × g for 5 min. Eluates were dried under vacuum using a SpeedVac (Thermo Fisher Scientific, Waltham, MA) for 1 h.

### Liquid chromatography–mass spectrometry

Peptides were reconstituted in 3% acetonitrile (ACN) with 0.1% formic acid (FA), and 150 ng of peptides were loaded per injection. Peptide separation was performed on a Vanquish Neo UHPLC system (Thermo Fisher Scientific) using an in-house packed analytical column (75 μm × 500 mm, 1.9 μm ReproSil-Pur 120 C18; Dr. Maisch GmbH, Germany). Mobile phase A consisted of 0.1% FA in water and mobile phase B consisted of 80% ACN with 0.1% FA. Peptides were eluted at a flow rate of 0.3 µL/min using a segmented gradient of 3% to 35% B over 66 min, 35% to 60% B over 2 min, and 60% to 98% B over 2 min, followed by washing at 98% B with flow increased from 0.3 to 0.5 µL/min over 5 min before re-equilibration at 3% B for 2.7 column volumes.

The LC system was coupled to a Thermo Fisher Scientific Orbitrap Eclipse mass spectrometer operated in positive ion mode with a spray voltage of 2.3 kV and ion transfer tube temperature of 300 °C. The Orbitrap Eclipse was operated in data-independent acquisition (DIA) mode with a normalised AGC target of 1000% and a maximum MS/MS injection time of 30 ms.

DIA data were acquired using sequential isolation windows spanning m/z 350–1650, with 20 variable-width overlapping windows. Survey scans were acquired at 30,000 resolution over m/z 350–1650 with a maximum injection time of 54 ms. Fragmentation was performed using stepped higher-energy collisional dissociation (HCD) at 25, 27, and 30 normalised collision energy (NCE), and MS/MS spectra were acquired at 30,000 resolution over m/z 300–2000. Bovine serum albumin (BSA) quality control runs confirmed consistent chromatographic performance and instrument stability, with retention time variation <0.1 minutes and sequence coverage of 38–42%.

### Proteomics processing

DIA data were analyzed using Spectronaut v20.1.250624 against the *Ovis aries* UniProt database (UP000002356_9940; release 2025_03) with trypsin specificity allowing up to two missed cleavages. Carbamidomethylation of cysteine was set as a fixed modification, with methionine oxidation and protein N-terminal acetylation set as variable modifications. Peptide and protein identifications were filtered to ≥ 95% confidence, with a minimum of two unique peptides required per protein.

DDA data were processed separately using Proteome Discoverer v2.5 with Mascot v3.1 (machine learning enabled) against the same database to generate complementary identification data. Comparative searches were also performed in Spectronaut using identical database parameters. Principal component analysis (PCA) and correlation analyses were performed to assess clustering and reproducibility.

### Differential analysis

Differential protein abundance analysis was performed using DIA-derived quantitative data based on normalised intensity values generated in Spectronaut. Comparative analyses were undertaken between whole seminal plasma (SP) and extracellular vesicle-depleted seminal plasma (DSP), extracellular vesicles (EV) and whole SP, and across sequential fractions (F1–F5). Protein abundance differences were evaluated using fold change analysis, with proteins exhibiting normalised intensity values ≥5 and a fold change ≥1.5 between groups considered for downstream interpretation.

### Functional annotation and network analysis

Proteomic datasets were analyzed using Ingenuity Pathway Analysis (IPA, Qiagen, Hilden, Germany) to identify enriched pathways. For all comparisons, a fold-change threshold of 1.5 (Log2FC ≥ 0.585 or ≤ -0.585) was applied to define proteins as up- or downregulated. Adjusted p-values were determined using Fishers exact testing with a significance cutoff of p = 0.05. A Venn diagram was generated to compare the protein composition protective SP treatments. Functional annotation clustering was performed in DAVID (54, 55), https://davidbioinformatics.nih.gov.

## Statistical analysis

All data were analyzed using R (version 2023.03.0). Linear mixed-effects models were fitted for each response variable, with sperm treatment included as the fixed effect and experimental replicate and ram included as random effects to account for repeated experimental variation and biological variability between males. Models were fitted using restricted maximum likelihood (REML), and degrees of freedom for fixed effects were estimated using Satterthwaite’s approximation. *Post hoc* pairwise comparisons were performed using estimated marginal means with Tukey adjustment for multiple comparisons. Statistical significance was set at *p* < 0.05. Data are presented as mean ± standard error of the mean.

### ResultsBoth extracellular vesicles and soluble proteins from seminal plasma contribute to protection against PMN activation and binding

**Figure 1:**
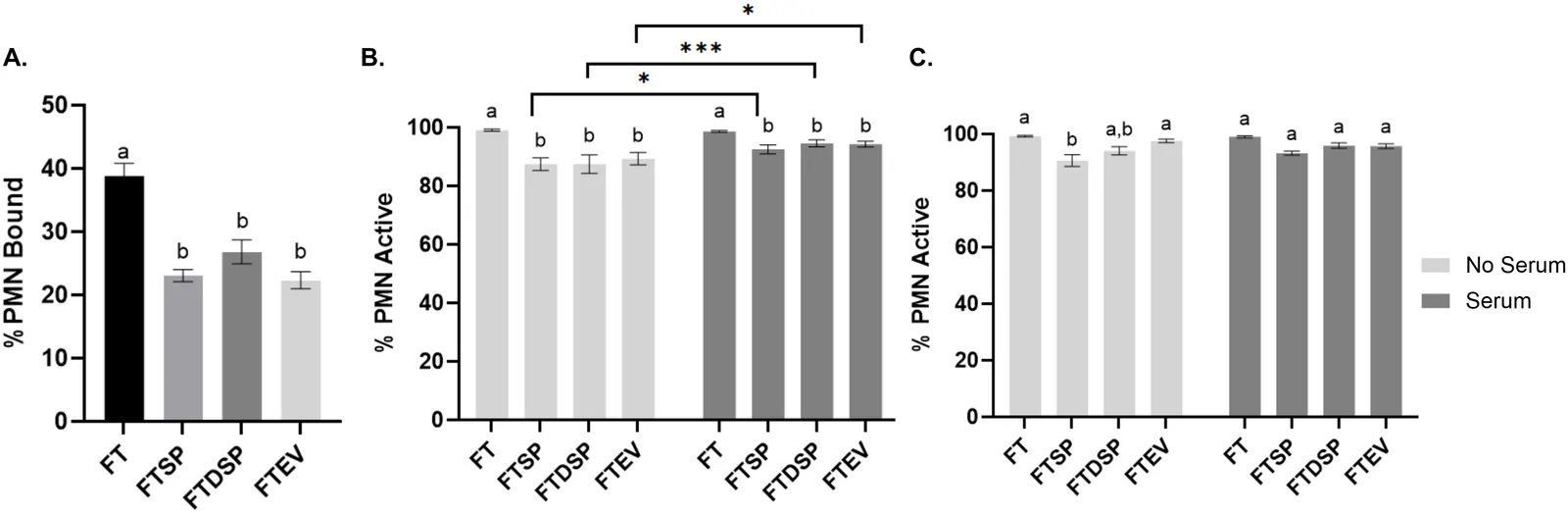
Polymorphonuclear neutrophil (PMN) responses to frozen-thawed ram spermatozoa under different seminal plasma treatments in Study 1. Spermatozoa were incubated with isolated PMNs under four seminal plasma (SP) treatments (1:1 v/v): frozen-thawed spermatozoa control (FT), whole SP supplemented (FTSP), SP depleted of extracellular vesicles (FTDSP), and supplemented with purified extracellular vesicles (FTEV). Experiments were conducted in the absence of serum (No Serum) and in 7.5% serum (Serum). Samples were assessed for PMN activation and binding at 60- and 120-minutes post incubation using Wright's staining solution. **(A)** Percentage of PMNs bound to ≥1 spermatozoon. Serum treatment and timepoint had no significant effect on binding; therefore, data were pooled across serum and time. **(B–C)** Percentage of activated PMNs at 60 and 120 minutes, respectively. Data are presented as mean ± SEM, averaged across rams (n = 3) and experimental replicates (n = 3). Statistical analysis was performed using linear mixed-effects models with Kenward–Roger degrees-of-freedom adjustment. For panel A, different= superscripts denote significant differences between SP treatments (Tukey-adjusted, p < 0.05). For panels B–C, different superscripts denote significant differences between treatments within each serum condition at a given timepoint (Tukey-adjusted, p < 0.05). Asterisks (*) indicate a significant effect of serum within a given treatment (p < 0.05).

Seminal plasma treatment of frozen-thawed spermatozoa had a significant effect on PMN binding (*p* < 0.0001). Estimated marginal means (averaged over Serum; ± SEM) resulted in FTSP (20.7 ± 3.39%), FTDSP (24.3 ± 3.64%) and FTEV (19.8 ± 3.63%) recording a significantly lower binding percentage compared to FT (36.3 ± 3.63%) (**Figure 1A**). No differences were observed between the treated groups (FTSP vs FTDSP: *p* = 0.407; FTSP vs FTEV: *p* = 0.975; FTDSP vs FTEV: *p* = 0.198). Neither serum (*p* = 0.616) nor timepoint (*p* = 0.116) significantly affected PMN binding, and variation between rams was negligible, indicating a consistent response across biological replicates.

PMN activation was significantly influenced by treatment (p < 0.0001), serum (p = 0.001), and timepoint (p = 0.00015), with a significant timepoint × serum interaction (p = 0.013). The treatment × serum interaction approached significance (p = 0.067), indicating that serum modulated the magnitude of treatment effects without fully abolishing them.

At 60 min in the absence of serum, PMN activation was significantly reduced in FTSP (87.44 ± 2.18%), FTDSP (87.44 ± 3.15%), and FTEV (89.33 ± 2.09%) compared with FT (99.06 ± 0.36%) (p < 0.0001 for all comparisons; **Figure 1B**). In the presence of serum, treatment effects were attenuated but not eliminated, with FTSP remaining significantly lower than FT (p = 0.0145), whereas differences between FT and FTDSP (p = 0.1839) and FT and FTEV (p = 0.1388) were no longer significant. Across serum conditions, serum significantly increased PMN activation in FTSP (p = 0.01), FTDSP (p < 0.001), and FTEV (p = 0.01), but had no effect on FT controls (p = 0.8246), indicating that serum selectively modulated treatment-associated suppression of activation.

At 120 minutes, PMN activation increased significantly from 60 minutes (*p* = 0.00015), but the overall pattern of treatment effects was maintained. In the absence of serum, PMN activation remained lower in FTSP (90.61 ± 2.06%; p < 0.0001), FTDSP (94.06 ± 1.45%; p = 0.001), and FTEV (97.56 ± 0.64%; p = 0.04) compared to FT (99.28 ± 0.29%) (**Figure 1C**) but the overall pattern of treatment effects was maintained.

### Moderate and high MW seminal plasma fractions reduced sperm-PMN binding and activation

PMN binding and activation were significantly affected by treatment (*p* < 0.0001 for both), with no significant interaction of treatment and timepoint (*p* > 0.05).

**Figure 2:**
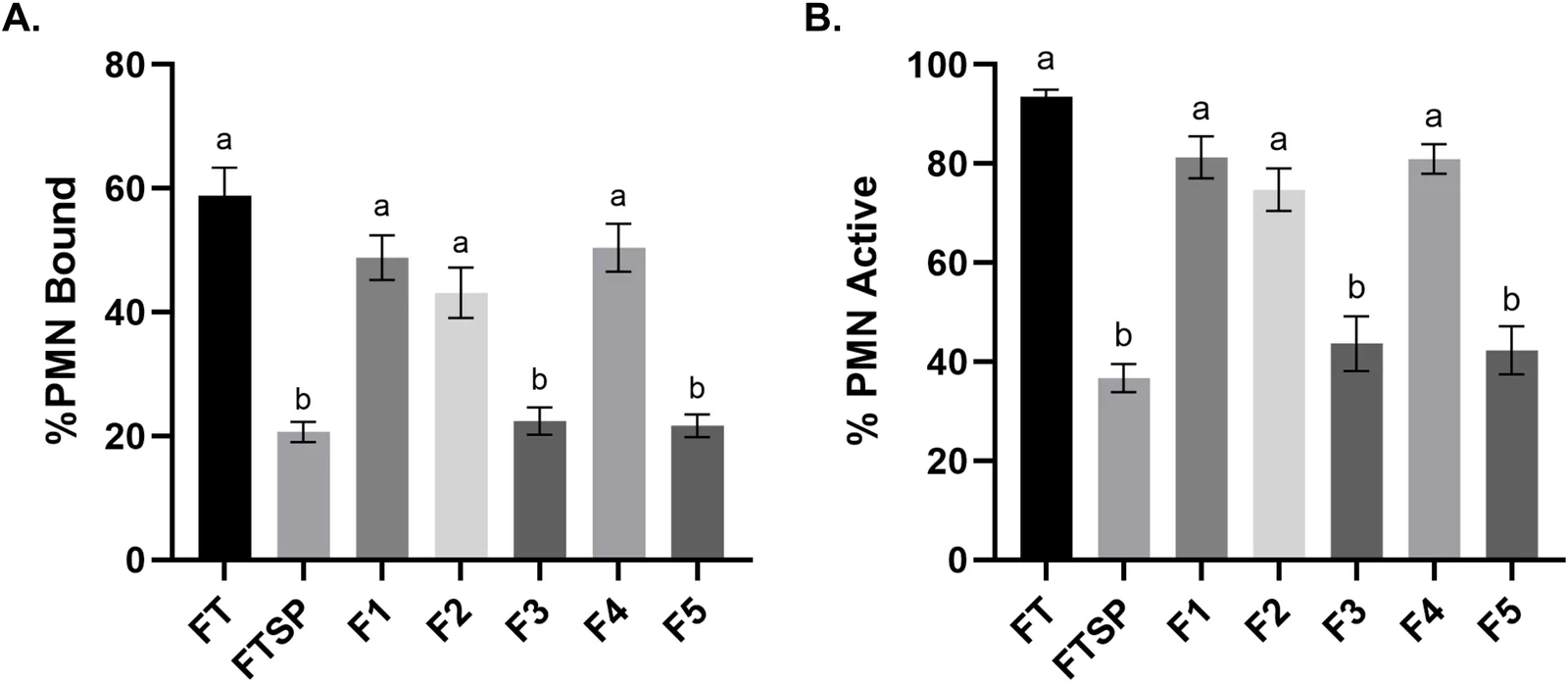
Response of polymorphonuclear neutrophils to frozen-thawed ram spermatozoa treated with seminal plasma fractions. Spermatozoa were incubated with isolated PMNs under 7 seminal plasma (SP) treatments (1:1 v/v): frozen-thawed sperm control (FT), whole SP supplemented (FTSP), and 5 MWCO fractions, F1 [<10 kDa], F2 [10–30 kDa], F3 [30–50 kDa], F4 [50–100 kDa], F5 [>100 kDa]. Data are presented as mean ± SEM, averaged across timepoint (60, 120mins), rams (n = 3) and replicates (n = 3). Samples were assessed for A) PMN binding and B) activation at 120 minutes using Wright's staining solution. Columns with different superscripts denote statistically significant differences (p < 0.05) between treatments.

FTSP, F3, and F5 consistently reduced both PMN binding (**Figure 2A**; 20.67 ± 1.65%, 22.44 ± 2.18%, 21.67 ± 1.85%, respectively) and activation (**Figure 2B**; 36.72 ± 2.83%, 43.67 ± 5.52%, 42.33 ± 4.87%, respectively) compared with the FT control (58.83 ± 4.54%; 93.50 ± 1.47%, respectively; *p* < 0.0001) (**Figures 2A, B**). There was no significant difference between FTSP, F3, and F5 (*p* > 0.05), nor between FT and F1, F2, and F4 (*p* > 0.05).

### Characterization of a homogeneous population of extracellular vesicles in Ram seminal plasma

**Figure 3:**
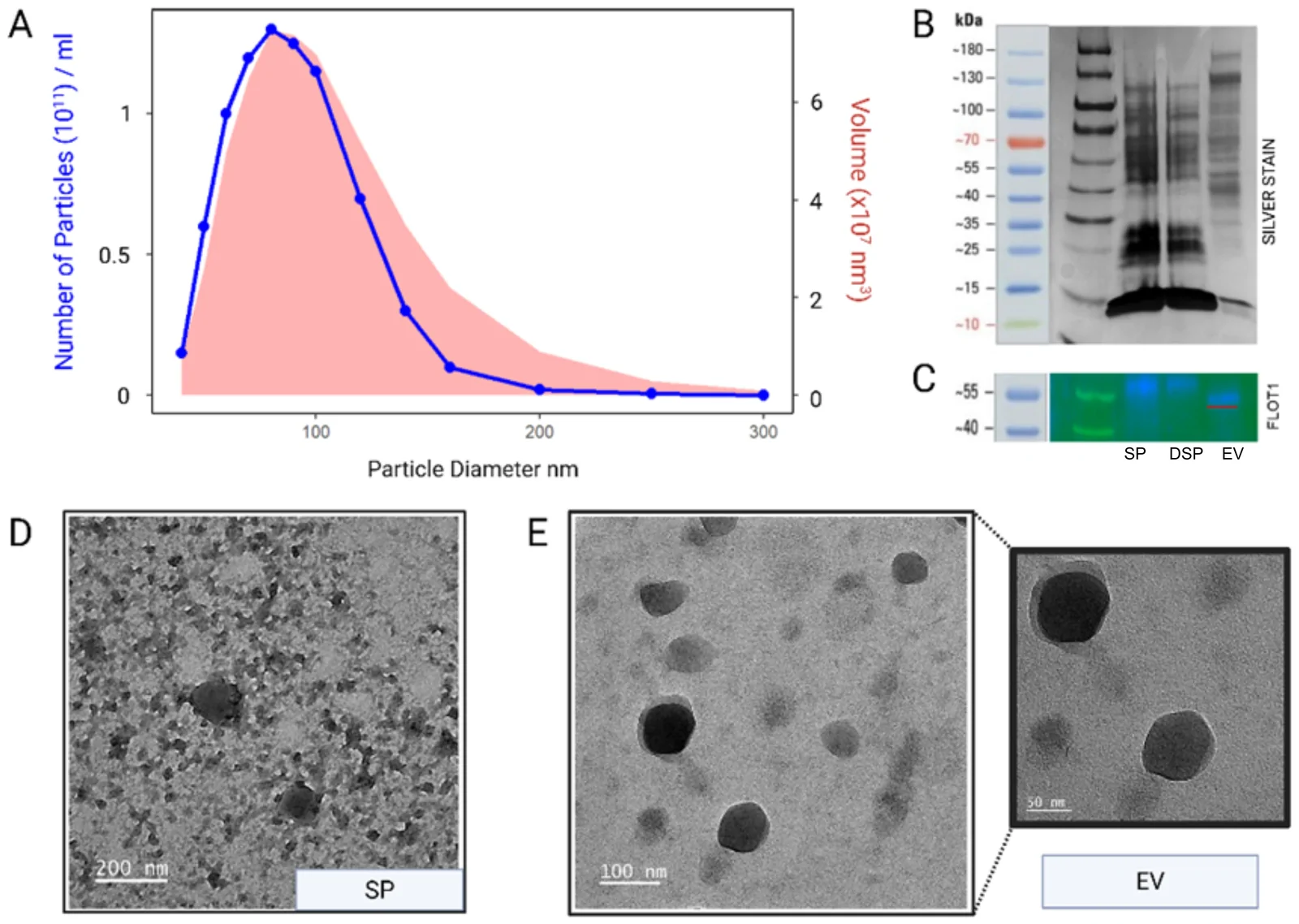
Characterisation of extracellular vesicles (EVs) enriched from ram seminal plasma. **(A)**. Particle size distribution of EVs, showing particle diameter (nm) relative to particle concentration (blue line with markers) and particle volume (red shaded ribbon). Concentration values represent the original (undiluted) sample and display a peak at 80 nm (~1.3 × 10^11^ particles/mL), indicating the most abundant vesicle population. The volume-weighted distribution is scaled for visual comparison; corresponding true volume values (nm³) are shown on the secondary y-axis. Median particle diameter (X50) is 93.6 nm (SD 49.4 nm). Correcting for a 1:10,000 dilution yields a total concentration of 1.9 × 10^12^ particles/mL. **(B)**. Silver stain confirming distinct protein profiles in whole seminal plasma (SP), EV-depleted seminal plasma (DSP), and purified EV fractions (EV). **(C)**. Immunoblot showing enrichment of the EV-associated marker flotillin-1 (FLOT1) in purified EVs. **(D, E)**. Transmission electron microscopy demonstrating vesicular morphology: **(D)** whole seminal plasma (25 kx) and **(E)** purified EVs (50 kx and 100 kx). Scale bars as indicated. All experiments were performed using pooled seminal plasma from n = 26 rams.

Extracellular vesicles (EVs) were successfully enriched from ram seminal plasma and characterized for size, morphology, and protein content (**Figure 3**). Nanoparticle tracking analysis revealed a predominantly homogenous population of small vesicles, with a peak concentration at 80 nm (~1.3 × 10¹¹ particles/mL), a median diameter (X50) of 93.6 nm (SD 49.4 nm), and a particle diameter range spanning X10 = 59.5 nm to X90 = 165.6 nm (**Figure 3A**), demonstrating a relatively narrow size distribution consistent with TEM observations (**Figures 3D, E**). The total particle concentration, following 1:10,000 dilution correction, was estimated at 1.9 × 10¹² particles/mL.

Protein characterization via silver staining demonstrated distinct patterns between whole seminal plasma (SP), EV-depleted SP (DSP), and purified EV fractions, indicating successful enrichment of vesicular proteins (**Figure 3B**). Western blotting confirmed EV-specific enrichment, with flotillin-1 (FLOT1) strongly detected in the purified EV fraction, supporting the purity of the preparation (**Figure 3C**). TEM imaging revealed round, membrane-bound vesicles of approximately 100 nm in diameter (**Figures 3D, E**). EV-like structures were present in SP samples (**Figure 3D**), however showed high soluble protein contamination compared to purified EV preparations, which depicted clear double membrane structures consistent with EVs (**Figure 3E**).

### Effect of seminal plasma treatment on the viability and motility of frozen-thawed spermatozoa and PMN viability

**Figure 4:**
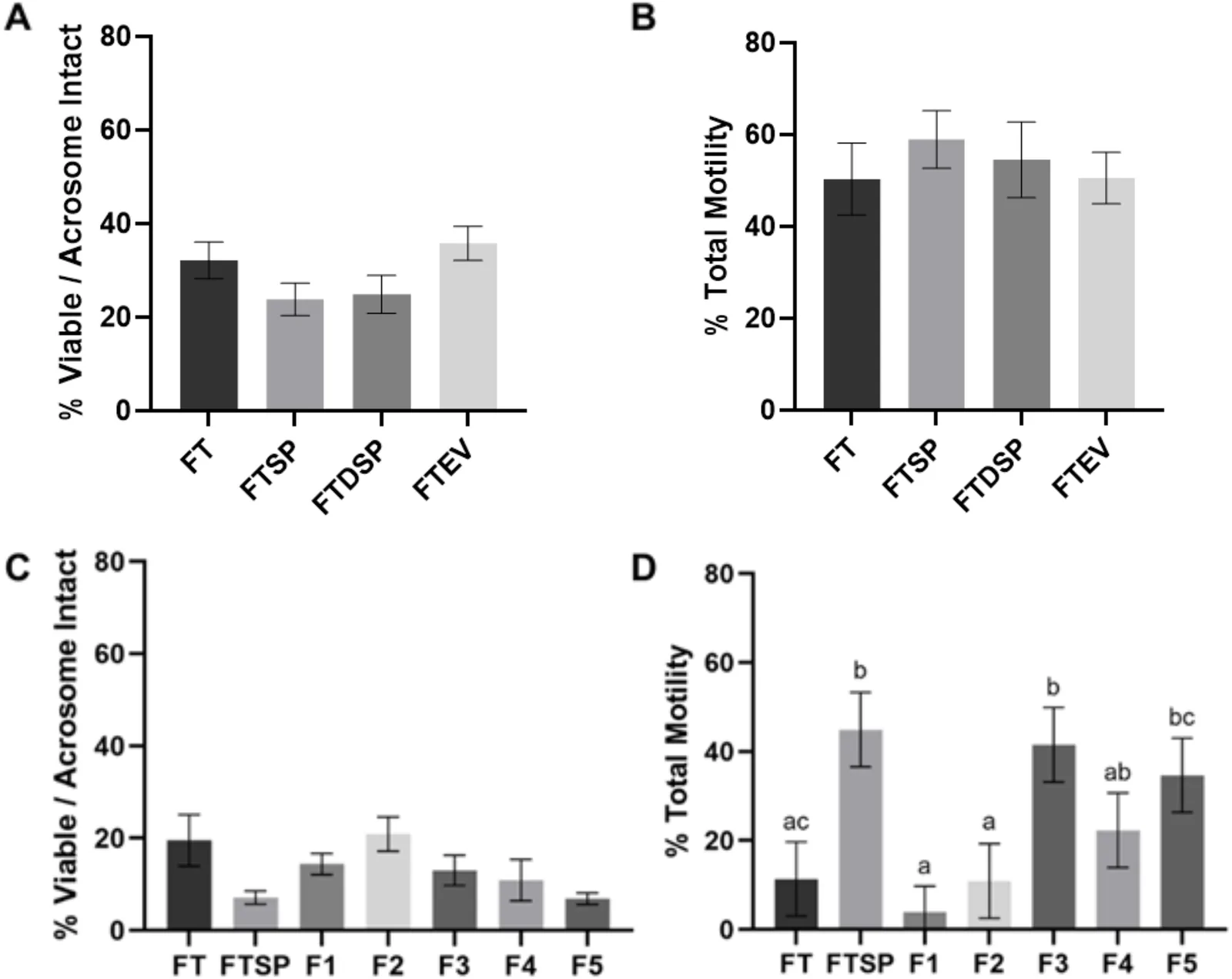
Effect of seminal plasma treatments on sperm viability and motility. In Study 1, Frozen-thawed spermatozoa were incubated under four treatments: frozen-thawed sperm control (FT), whole seminal plasma supplemented (FTSP), extracellular vesicle-depleted SP (FTDSP), and enriched EVs (FTEV). Data are presented as mean ±± SEM, averaged across rams (n = 3) and replicates (n = 3). Spermatozoa were assessed for **(A)** viability (VAI%) using flow cytometry and **(B)** total motility using CASA. No pairwise comparisons reached significance after Tukey adjustment; superscripts are therefore not shown. In Study 2, Frozen-thawed spermatozoa were incubated under seven treatments; FT control, FTSP, or one of five MWCO seminal plasma fractions: <10 kDa (F1), 10–30 kDa (F2), 30–50 kDa (F3), 50–100 kDa (F4), and >100 kDa (F5). Data are presented as mean ± SEM, averaged across rams (n = 3) and replicates (n = 3). Sperm were assessed for **(C)** viability (VAI%) and **(D)** total motility. Columns with different superscripts denote statistically significant differences (p < 0.05) based on Tukey-adjusted pairwise comparisons; only motility comparisons were significant.

In Study 1, spermatozoa viability (VAI%) differed significantly among treatments in the linear mixed model (*p* = 0.0136); however, following Tukey adjustment for multiple comparisons, no individual treatment pair differed significantly (**Figure 4A**; values in **Supplementary Table 2**). Total motility as measured by CASA did not differ between treatments (*p* = 0.7903; **Figure 4B**).

In study 2, treatment showed a significant overall effect on VAI% in the model (*p* = 0.0357), but no pairwise comparisons remained significant after Tukey adjustment (**Figure 4C**; values in **Supplementary Table 3**). In contrast, total motility as assessed by CASA was significantly affected by treatment (*p* < 0.001; **Figure 4D**). Pairwise comparisons revealed Motility was significantly higher in FTSP (44.93 ± 5.90%, *p* = 0.0179) and F3 (41.55 ± 5.90%, *p* = 0.0481) relative to the FT control (11.37 ± 8.35%). F5 (38.57 ± 5.90%) and F4 (26.18 ± 5.90%) showed a moderate increase compared to FT but did not reach statistical significance (*p* < 0.05). Notably, both F4 and F5, although not significantly different from FT, were also not significantly different from the higher-motility groups FTSP and F3, suggesting intermediate motility levels relative to the low (FT, F1, F2) and high-motility (FTSP, F3) treatments.

Treatment had no significant effect on PMN viability (*p* > 0.05). In both studies, the percentage of viable PMNs remained high at 0 minutes and declined similarly after 120 minutes. In Study 1, average viability across treatments was 97.3 ± 0.7% at 0 minutes and 82.0 ± 1.5% at 120 minutes. In Study 2, average viability was 92.2 ± 1.3% at 0 minutes and 80.0 ± 1.5% at 120 minutes (**Supplementary Table 4**).

### Protective Ram SP fractions share a unique core subset of proteins

LC–MS/MS allowed for the identification of a total of 2,161 proteins in whole ram seminal plasma (**Supplementary Table 5**). Of these, 1,827 could be mapped to known gene names. Notably, ram SP-EVs contained 795 proteins not contained within the SP dataset, with 700 annotated to known genes. Across all sample types analyzed by DIA (From study 1 and 2), a total of 3,026 proteins were identified. Protein identifications were supported by 2–177 peptides per protein (mean 9.2), with 87.1% of proteins supported by ≥2 peptides.

Fractionation of SP based on molecular weight in study 2, revealed that whole SP contained the largest number of unique proteins (87), followed by the >100 kDa fraction (F5; 43). Smaller numbers of unique proteins were detected in other fractions: <10 kDa (F1; 4), 10–30 kDa (F2; 6), 30–50 kDa (F3; 34), and 50–100 kDa (F4; 3). Across all fractions, 55 proteins were shared (**Supplementary Table 6**).

The top 10 most abundant proteins across all SP-derived samples (Study 1 and Study 2) are summarized in **Table 1**, including SP, DSP, EV, and the molecular weight fractions F1–F5.

**Table 1:** Summary of the top 10 most abundant proteins quantified across SP, DSP, EV, and isolated fractions; <10 kDa, 10–30 kDa, 30–50 kDa, 50–100 kDa, and >100 kDa, using data-independent acquisition (DIA) proteomics.

Protein	SP	DSP	EV	F1	F2	F3	F4	F5
LOC101113728	22.71	22.56		16.36				22.61
W5P1P8	22.58	21.98		18.25			21.25	22.35
BSP5	21.84	22.81			22.22	23.58	23.53	22.77
RNASE9	21.32	21.26					21.72	21.10
MSMB	21.11	20.78			24.24	23.69	22.04	
EDIL3	21.10		23.49	16.99				21.13
LOC101116661	21.09	21.34						21.18
LOC101116409	20.95	20.99						21.00
PYY	20.91	21.17						21.28
W5PE35	20.87			15.90				
CES5A		20.81						20.74
ACE			23.46	15.99				
PROM2			23.12					
ENPP3			23.02					
CD9			22.84					
MME			22.54					
ELSPBP1			22.46					
ACTB			22.06	15.83				
RAC3			22.04	16.47				
W5NQA4			21.56					
NPPC				19.51	21.91			
WFDC2					24.07	23.80	21.85	
W5P886					22.83	22.55		
NPC2					22.56	22.46	21.48	
rsvp14					21.15	21.69		20.71
W5NUE4					21.14	22.65	21.62	
SOD1					20.98	21.29		
CST6					20.45			
ALB							21.32	
LOC101111505		20.75		15.90			21.00	
LOC101110099						21.68	21.39	

Protein abundance is shown as log2-transformed expression values for direct comparison across sample types.

**Figure 5:**
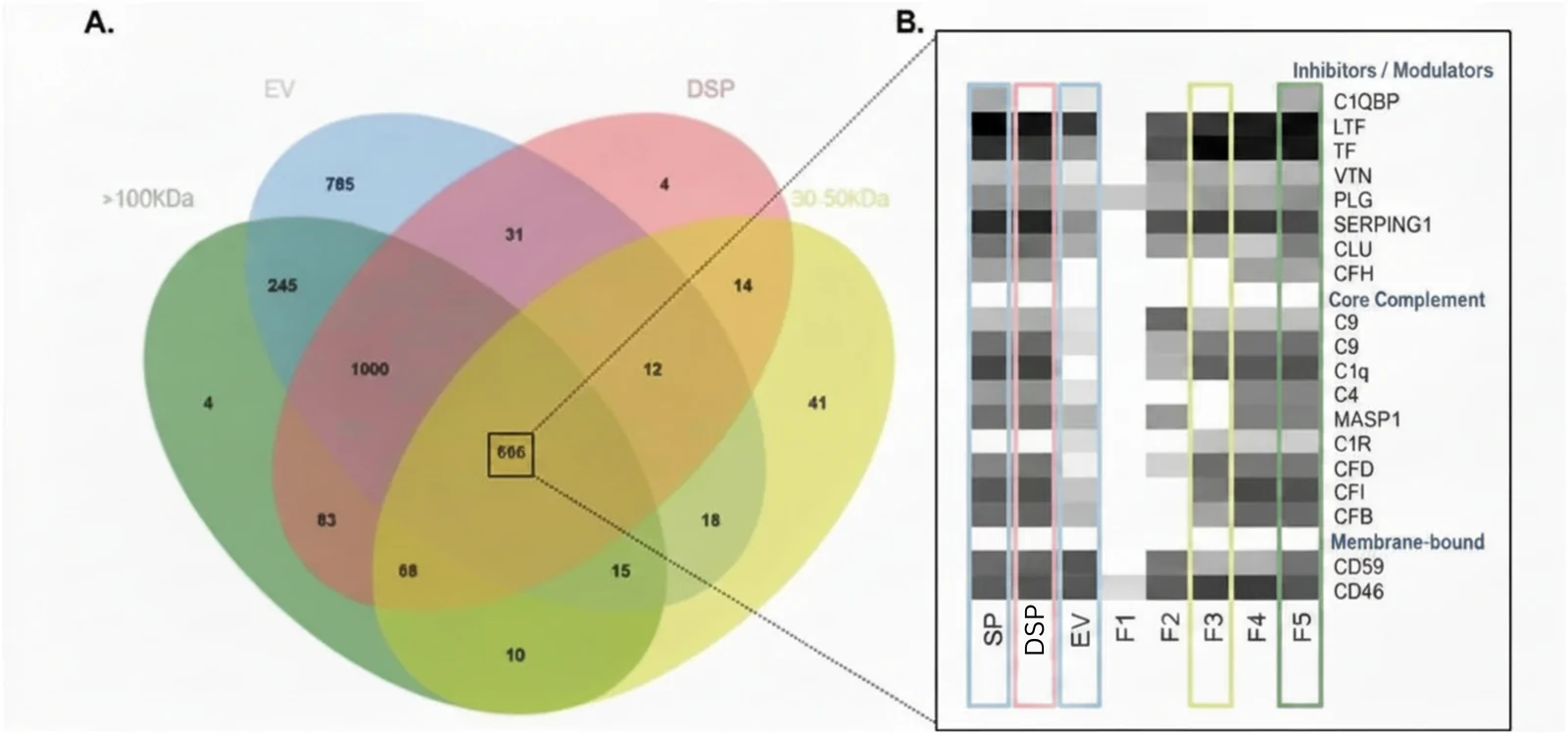
Unique proteins present in seminal plasma treatments that had a protective effect against PMN binding. **(A)** Shared protein expression of protective fractions. DIA protein lists for each treatment were compared in R, with proteins present in protective treatments; whole seminal plasma (SP), extracellular vesicle-depleted SP (DSP), extracellular vesicles (EV), 30-50kDa fraction (F3) and >100kDa fraction (F5) compared. **(B)** Expression of Complement proteins and regulators. The figure includes a key panel displaying identified complement factors, including soluble inhibitors and modulators, core complement components, and membrane-bound regulators, demonstrating their distribution across all SP-derived samples and emphasising the multi-layered molecular contributions to PMN inhibition.

Subsequent analysis focused on the protective seminal plasma fractions (SP, DSP, EV, F3, F5). Among these, 588 proteins were shared across all four protective fractions (**Figure 5A**), 373 of which were mapped to known genes, including ALB, AHSG, DEFB124, CRISP3, PTGDS, and B2M. Seven proteins were uniquely detected in the protective fractions and absent from non-protective fractions (F1, F2, F4): PITPNB, RPLP2, SLC9A3R1, PTTG1IP, CKAP4, FKBP11, and W5PXT2 (unannotated). Amongst the proteins shared across protective fractions, a number of known immune regulatory factors were present. These included soluble complement modulators Complement Factor H (CFH), Clusterin (CLU), core proteins C3, C9, C4 and membrane bound complement regulatory proteins CD46 and CD59 (**Figure 5B**).

Of the proteins detected in the protective fractions, EVs contributed a substantial set of unique proteins (785), while the majority of proteins were shared among EV, DSP, and >100kDa (1,008). Additional overlaps included EV and >100kDa (245 proteins), EV and DSP (31 proteins), and DSP with >100kDa (83 proteins) (**Figure 5A**).

### Functional enrichment of seminal plasma and fractionated proteins reveals distinct innate immune signatures

DAVID functional annotation clustering was performed to characterize the protein composition of seminal plasma (SP) and fractionated samples, comparing PMN-binding protective (SP, EV, DSP, F3, F5) and non-protective fractions (F1, F2, F4). Enrichment patterns were broadly dominated by extracellular localization, innate immune functions, and vesicle/transport-associated processes, with distinct differences between protective and non-protective fractions (**Supplementary Table 7**).

SP displayed the strongest overall enrichment for extracellular and secreted proteins, with Cluster 1 (enrichment score = 9.05) dominated by proteins annotated as *Secreted* (p = 7.72 × 10^-13^), *extracellular region* (p = 5.34 × 10^-10^), and *Signal* domain-containing proteins (p = 1.72 × 10^-6^). Immune-associated enrichment was prominent in Cluster 2 (enrichment score = 4.65), which included *antimicrobial* (p = 7.05 × 10^-7^), *defensin* (p = 4.18 × 10^-7^), and *antibiotic* (p = 1.63 × 10^-8^) annotations, together with *innate immune response* (p = 0.0012), *defense response to bacterium* (p = 1.35 × 10^-5^), *chemoattractant activity* (p = 4.35 × 10^-6^), *CCR6 chemokine receptor binding* (p = 2.07 × 10^-5^), and *cell chemotaxis* (p = 0.00111). Additional immune-relevant annotations included *lipopolysaccharide binding* (Cluster 3; enrichment score = 4.44; p = 1.38 × 10^-4^). Collectively, SP exhibited broad enrichment for extracellular secreted proteins and innate immune-associated functional categories, including antimicrobial, chemotactic, and bacterial defense pathways.

EVs and F5 showed highly similar enrichment profiles characterized by strong extracellular localization, innate immune-associated functions, and the presence of complement-associated Sushi/CCP domain architecture. In EVs, Cluster 1 (enrichment score = 6.67) was dominated by *Secreted* (p = 8.78 × 10^-10^) and *extracellular region* (p = 3.7 × 10^-7^) annotations. Immune-related enrichment was observed in Cluster 2 (enrichment score = 3.98), including *antimicrobial* (p = 1.25 × 10^-6^), *defensin* (p = 6.77 × 10^-7^), *chemoattractant activity* (p = 7.99 × 10^-6^), *CCR6 chemokine receptor binding* (p = 3.27 × 10^-5^), and *innate immune response* (p = 0.00292), together with bacterial defense pathways such as *defense response to bacterium* (p = 3.14 × 10^-5^). EVs also showed enrichment of vesicle-associated processes including *phagocytic vesicle membrane* (p = 0.0269). Importantly, EVs additionally exhibited enrichment for Sushi/CCP domain-containing proteins (Cluster 9; enrichment score = 1.43; p = 0.0316).

F5 (>100 kDa) demonstrated a comparable profile with Cluster 1 (enrichment score = 7.35) enriched for *Secreted* (p = 8.88 × 10^-13^), *extracellular region* (p = 1.41 × 10^-9^), and *Signal* domains. Immune-related enrichment in Cluster 2 (enrichment score = 4.97) included *defensin* (p = 2.94 × 10^-7^), *chemoattractant activity* (p = 3.24 × 10^-6^), *CCR6 chemokine receptor binding* (p = 1.66 × 10^-5^), and *defense response to bacterium* (p = 9.96 × 10^-6^). F5 also showed enrichment of *lipopolysaccharide binding* (Cluster 4; enrichment score = 4.57; p = 1.11 × 10^-4^) and contained Sushi/CCP domain-associated annotations (Cluster 9; enrichment score = 1.43; p = 0.0316). Overall, EVs and F5 exhibited highly concordant enrichment of extracellular proteins and innate immune-associated pathways, including antimicrobial, chemotactic, and bacterial defense functions.

DSP and F3 shared a core enrichment profile characterized by extracellular localization and antimicrobial/innate immune functions, though with a comparatively narrower pathway distribution than SP, EVs, and F5. DSP showed strong enrichment for secreted proteins in Cluster 1 (enrichment score = 6.99), including *Secreted* (p = 9.89 × 10^-13^) and *extracellular region* (p = 3.71 × 10^-9^). Immune-related enrichment in Cluster 2 (enrichment score = 3.58) included *antimicrobial* (p = 4.16 × 10^-7^), *defensin* (p = 1.23 × 10^-5^), *innate immune response* (p = 0.0224), and *defense response to bacterium* (p = 1.68 × 10^-4^), along with *lipopolysaccharide binding* (p = 3.62 × 10^-^³).

F3 showed a similar pattern, with Cluster 1 (enrichment score = 10.95) strongly enriched for *Secreted* (p = 1.03 × 10^-^¹^4^) and *extracellular region* (p = 6.33 × 10^-^¹²). Immune-related enrichment included *antimicrobial* (p = 1.41 × 10^-5^), *innate immune response* (p = 0.014), and *defense response to Gram-positive and Gram-negative bacterium* (p = 1.97 × 10^-4^; p = 0.00311). F3 additionally showed enrichment for the *NOD-like receptor signaling pathway* (p = 8.98 × 10^-5^). Together, DSP and F3 retained enrichment for extracellular antimicrobial and innate immune-associated pathways, with F3 showing additional enrichment of intracellular innate immune signaling (NOD-like receptor pathway).

In the non-protective fractions (as determined by PMN-binding assay) F1 showed no significant functional enrichment clusters, indicating a lack of coordinated over-representation of annotated biological processes. F2 (10–30 kDa) displayed strong enrichment for extracellular and secreted proteins in Cluster 1 (enrichment score = 8.57), including *Secreted* (p = 4.99 × 10^-13^) and *extracellular region* (p = 2.14 × 10^-^¹¹). Immune-related enrichment in Cluster 2 (enrichment score = 3.36) included *antimicrobial* (p = 8.82 × 10^-6^), *innate immune response* (p = 0.0111), and *NOD-like receptor signaling pathway* (p = 3.81 × 10^-5^), together with bacterial defense annotations such as *defense response to bacterium* and *Staphylococcus aureus infection*. However, chemotaxis-related pathways and Sushi/CCP domain enrichment were not prominent in this fraction. F4 (50–100 kDa) exhibited strong enrichment for secreted and extracellular proteins in Cluster 1 (enrichment score = 8.27), including *Secreted* (p = 1.18 × 10^-^¹^4^) and *extracellular region* (p = 2.13 × 10^-9^). Immune-associated enrichment in Cluster 4 (enrichment score = 2.61) included *antimicrobial* (p = 0.00112), *lipopolysaccharide binding* (p = 0.00176), and *innate immune response* (p = 0.0351). F4 additionally showed enrichment for the *NOD-like receptor signaling pathway* (Cluster 5; enrichment score = 2.38; p = 5.6 × 10^-5^) alongside signaling-associated pathways such as PI3K-Akt and Hippo signaling. Overall, the non-protective fractions retained extracellular and antimicrobial-associated annotations; however, enrichment was less consistently associated with chemotactic pathways and complement-associated Sushi/CCP domain architecture compared with protective fractions.

### Pathway enrichment of EV and EV depleted seminal plasma proteins reveals contrasting immune signatures

Differential expression analysis identified 1,797 proteins in EV versus SP, of which 1,370 were upregulated and 427 downregulated, and 479 proteins in DSP versus SP, including 78 upregulated and 401 downregulated. Further 1,199 Proteins were shared between EVs and DSP (**Supplementary Table 8**). While, canonical pathway analysis using IPA revealed a clear divergence between EV and DSP, particularly in immune-related signaling.

**Figure 6:**
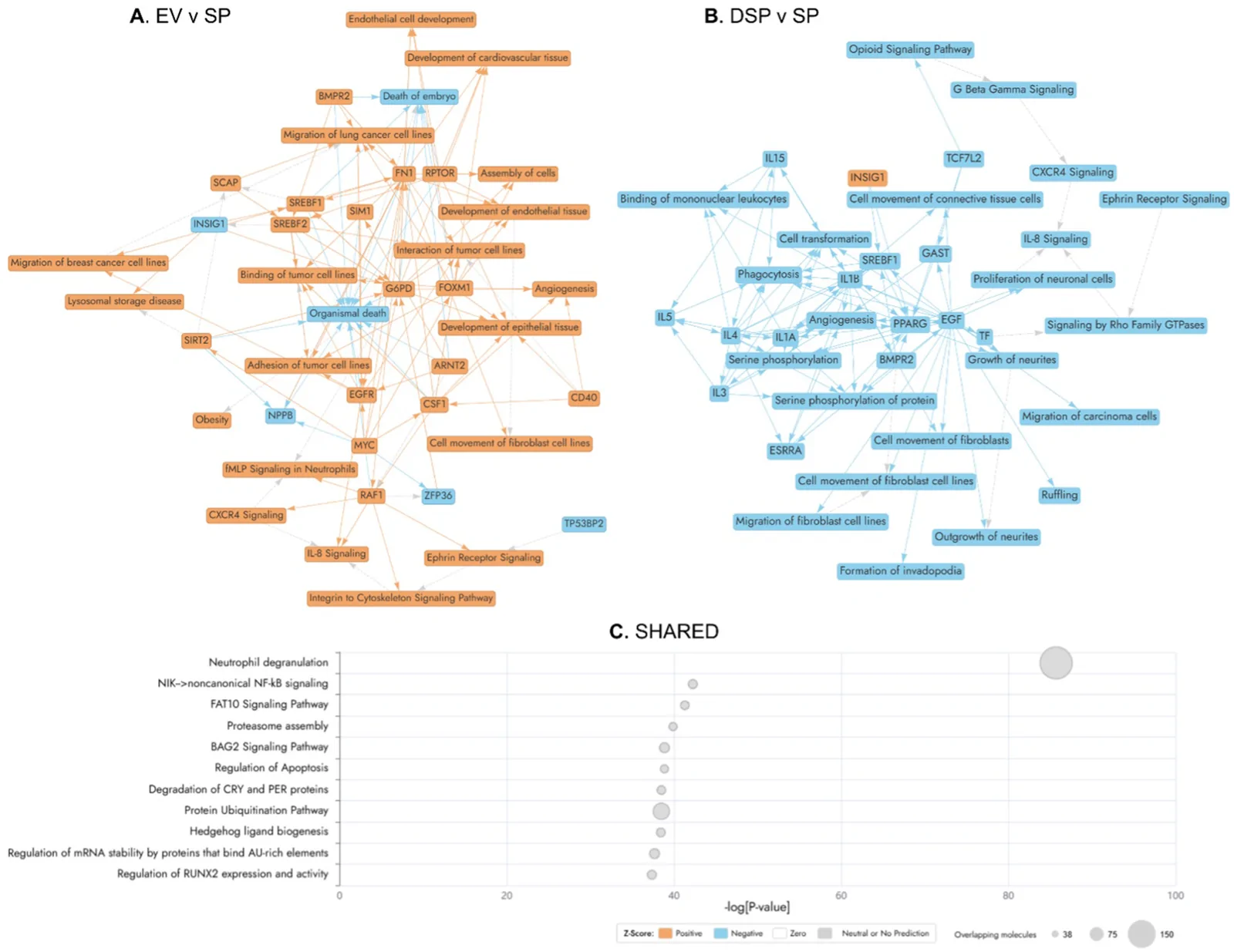
Comparison of canonical pathways identified by IPA for extracellular vesicles (EV) and extracellular vesicle–depleted seminal plasma (DSP). **(A)**. For the EV vs SP contrast, 1,797 proteins met differential expression cutoffs (log₂ fold change < –0.585 or > 0.585), with 427 downregulated and 1,370 upregulated. **(B)**. For the DSP vs SP comparison, 479 proteins passed the same cutoffs, comprising 401 downregulated and 78 upregulated proteins. Predicted activation is depicted in orange, while predicted inhibition is depicted in blue, as per figure legend. **(C)**. For proteins present in both EV and DSP data, 1,199 proteins met differential expression cutoffs (log₂ fold change < – 0.585 or > 0.585), with representative conserved pathways derived from shared list shown.

EVs exhibited strong predicted activation of multiple immune and cell signaling pathways (**Figure 6A**). The most significantly activated canonical pathways included Ephrin receptor signaling (z = 7.30, −log_10_p = 34.0) and CXCR4 signaling (z = 6.49, −log_10_p = 29.87). Additional enriched pathways included RHO GTPase cycle signaling (z = 9.52, −log_10_p = 31.60), IL-8 signaling (z = 5.70, −log_10_p = 31.70), fMLP signaling in neutrophils (z = 6.40, −log_10_p = 27.53), RAC signaling (z = 6.86, −log_10_p = 27.77), and actin cytoskeleton signaling (z = 6.20, −log_10_p = 25.19). Pathways associated with cell adhesion and junction dynamics were also enriched, including integrin signaling to cytoskeleton (z = 6.75, −log_10_p = 27.63) and epithelial adherens junction signaling (z = 2.72, −log_10_p = 25.14). Together, EV-associated pathways were strongly enriched for signaling networks involved in immune cell communication, chemotaxis-associated signaling, and cytoskeletal regulation, all of which contribute to neutrophil migration, adhesion and activation (**Supplementary Table 9**).

In contrast, DSP demonstrated predicted inhibition of multiple innate immune and chemotactic signaling pathways (**Figure 6B**). The most strongly inhibited pathways included neutrophil degranulation (z = −5.69, −log_10_p = 20.79), IL-8 signaling (z = −5.00, −log_10_p = 22.18), fMLP signaling in neutrophils (z = −4.58, −log_10_p = 25.16), and CXCR4 signaling (z = −4.58, −log_10_p = 25.16). Additional inhibited pathways included CCR3 signaling in eosinophils (z = −3.74, −log_10_p = 22.73), RAC signaling (z = −3.90, −log_10_p = 19.71), and Rho family GTPase signaling (z = −3.90, −log_10_p = 19.71). The predicted inhibition of IL-8 signaling, neutrophil degranulation and fMLP signaling is consistent with suppression of neutrophil activation and effector responses, supporting the reduced PMN activation observed in the functional assays (**Supplementary Table 10**).

IPA of the shared EV and DSP proteome identified strong enrichment of immune regulatory and cellular stress-response pathways. The most significantly enriched pathway was neutrophil degranulation (p = 1.96 × 10^-86^). Additional highly enriched canonical pathways included NIK-mediated non-canonical NF-κB signaling (p = 5.71 × 10^-43^), FAT10 signaling (p = 5.06 × 10^-4^²), proteasome assembly (p = 1.3 × 10^-40^), and regulation of apoptosis signaling (p = 1.42 × 10^-39^). Further enriched pathways included protein ubiquitination (p = 3.26 × 10^-39^), regulation of mRNA stability via AU-rich element binding proteins (p = 2.17 × 10^-38^), KEAP1–NFE2L2 signaling (p = 2.86 × 10^-32^), and class I MHC-mediated antigen processing and presentation (p = 8.25 × 10^-30^). Collectively, the shared proteome was strongly enriched for immune effector processes, protein turnover, inflammatory signaling regulation, and antigen processing pathways (**Figure 6C**; **Supplementary Table 11**).

## Discussion

The protective effect of seminal plasma on sperm–polymorphonuclear neutrophil (PMN) binding can be localized to discrete molecular fractions. Here, we demonstrate that this immunomodulatory activity is concentrated within extracellular vesicles (EVs) and defined protein subsets, namely the 30–50 kDa (F3) and >100 kDa (F5) fractions, which significantly reduced PMN-sperm binding. As EV preparations were validated and had no effect on PMN viability, the observed differences are likely attributable to specific immunoregulatory cargo rather than nonspecific effects. Protective SP treatments were characterized, revealing a conserved core protein signature of PITPNB, RPLP2, SLC9A3R1, PTTG1IP, CKAP4, and FKBP11, whose predicted functions suggest a structured SP-derived immunoregulatory system that selectively protects sperm against PMN while maintaining innate immune functionality. By integrating EV characterization, proteomics, and functional assays, this study demonstrates that inhibition of sperm–PMN binding is mechanistically distinct from complement-amplified PMN activation, indicating multiple layers of innate immune regulation. Complement emerges as a central axis, with protective fractions enriched in male-derived regulators including CD59, CD46, FH and CLU (**Figure 5**). The enrichment of immune-signaling proteins within EVs further supports their role as membrane-bound delivery systems capable of modulating neutrophil adhesion and inflammatory signaling. Seminal plasma additionally contains key modulators of the sperm glycocalyx and activates ubiquitin–proteasome networks, potentially remodeling sperm antigenicity and extending immune regulation toward adaptive tolerance.

Following insemination, the female reproductive tract mounts a rapid but transient inflammatory response in which PMNs balance pathogen clearance with sperm transport through tightly regulated molecular interactions (1, 36). Cryopreserved spermatozoa exhibit an altered molecular phenotype that may disrupt this immune equilibrium, whereas seminal plasma has been proposed to attenuate PMN activation by modulating sperm surface epitopes and neutrophil effector functions (6, 42). In the present study, whole SP robustly suppressed sperm–PMN binding, consistent with previous ovine work demonstrating effective SP-mediated shielding of frozen-thawed sperm from neutrophil recognition and binding (38, 56). Critically, this protective effect was reproduced by both DSP (soluble proteins) and enriched EVs, indicating that both soluble and vesicle-associated components contribute to sperm immune protection. Although soluble seminal plasma proteins, including DNase, CRISP3 and lactoferrin, have previously been associated with reduced PMN susceptibility and NETosis in equine and bovine models (39, 40, 57–59), this is the first study to functionally link ram seminal plasma EVs with protection against excessive PMN binding. This supports the concept that sperm immune protection is mediated by coordinated actions of multiple seminal plasma proteins rather than a single immunomodulator.

Fractionation by molecular weight demonstrated that only the 30–50 kDa (F3) and >100 kDa (F5) fractions effectively inhibited sperm–PMN binding, whereas low- and mid-molecular-weight fractions (<10 [F1], 10–30 [F2], 50–100 kDa [F4]) had no discernible effect. The restriction of activity to defined molecular windows is supported by previous work in the donkey (51) and suggests that sperm immunoprotection is not mediated by generalized protein abundance, but rather by specific protein factors capable of modifying the sperm surface or directly engaging neutrophil receptors. Together, these findings support a model in which seminal plasma delivers layered immunoregulatory cues, via both vesicular and soluble components, to sculpt a transiently tolerated sperm phenotype within the female tract. Leading to a focus on isolating the molecular candidates responsible for physiological effects *in vitro* through proteomic investigation.

A comparative analysis of protective seminal plasma (SP) fractions (DSP, EV, F3, F5) identified a conserved core of 588 proteins, with six uniquely shared: PITPNB, RPLP2, SLC9A3R1, PTTG1IP, CKAP4, and FKBP11. These proteins were absent from non-protective fractions and were associated with reduced PMN binding when supplemented to frozen-thawed spermatozoa, suggesting a minimal SP-derived protective signature. Analysis of published seminal plasma proteomes from ram, bull, boar, buck, stallion, alpaca, and camel identified RPLP2 and SLC9A3R1 (NHERF1) in ram seminal plasma, with SLC9A3R1 also reported in porcine seminal plasma as the PDZ1 domain, suggesting conservation across species (60–63). In contrast, the absence of PITPNB, PTTG1IP, CKAP4, and FKBP11 from previous datasets likely reflects limitations in proteome coverage and annotation rather than true species-specific absence, highlighting these proteins as potential targets for future investigation. While sperm-surface expression of these proteins was not assessed in the present study, cross-species data from the ShinySpermKingdom database (64) confirm that all 6 core proteins are present in the sperm proteomes of the mouse and human, with RPLP2 and PTTG1IP detected in bull, and PTTG1IP predicted in ram, suggesting they may be incorporated into the sperm proteome. Collectively, these proteins converge on pathways regulating endoplasmic reticulum stress, membrane organization, inflammatory signaling and immune cell recruitment, suggesting that sperm protection is mediated through coordinated regulation of cellular stress responses rather than a single immunomodulatory pathway (65). FKBP11 and CKAP4, for example, have recognised roles in limiting ER stress and inflammatory signaling, providing biologically plausible mechanisms through which seminal plasma may promote sperm survival within the post-insemination inflammatory environment (66–68).

Other members of the core set implicate regulated, rather than simply suppressed, innate immune activation. RPLP2 has been described as an extracellular ligand for TLR4, capable of amplifying NF-κB signaling, upregulating pro-inflammatory cytokines like TNF-α, IL-6, and IL-8 (69). In the reproductive landscape, TLR4 signaling has previously been described as a core mediator of female immune responses to seminal fluid in mice, induced expression of key proinflammatory cytokines and chemokines, including CSF3, CXCL1, CXCL2, IL1A, IL6, LIF, and TNF (70). Similarly, SLC9A3R1 (NHERF-1) participates in ICAM-1/ERM complex formation and pro-inflammatory signaling (71, 72). While seemingly counterintuitive, transient activation of these pathways may facilitate antigen sampling and immune priming, processes required for downstream tolerance induction (16). PTTG1IP may similarly contribute to this balance. Although currently supported only by indirect evidence, its association with TGF-β-related immune regulation and leukocyte recruitment suggests a potential role in coordinating early inflammatory responses with subsequent tolerogenic adaptation (16, 73). PITPNB also has an interesting link with PMNs, with this protein family acting as a crucial cofactor and regulatory protein in Phospholipase C (PLC) signaling (74). Its beta isoform, identified in the present study, predominates in neutrophils and may facilitate PI3Kγ-dependent PIP3 production following FMLP receptor engagement, enhancing neutrophil activation, chemotaxis, and responsiveness (75, 76). The presence of this key isoform in ram seminal plasma could position PITPNB as a regulator of sperm-PMN interactions, whether this action would be mediated on the sperm surface, or indirectly through delivery of this protein to the local female environment following insemination requires further investigation.

The enrichment of immune-associated proteins across protective seminal plasma fractions supports a model in which sperm immune protection arises through the coordinated actions of multiple proteins rather than individual effectors. Although a conserved subset was consistently associated with reduced PMN binding, these candidates are unlikely to act in isolation. Instead, proteins present in both soluble and EV-associated pools may operate through complementary mechanisms: soluble factors likely act rapidly within the female reproductive tract to modulate the immediate post-insemination inflammatory response, whereas EV-associated proteins provide more sustained signaling and may remodel sperm surface immune architecture through direct cell–cell communication. Differences in protein abundance and compartmentalization are therefore likely to shape functional outcomes. Targeted quantitative proteomic and localization studies will be essential to define the relative contribution of individual proteins and their interactions at the sperm surface.

Our functional data support a temporally and context-dependent immunoregulatory system, in which seminal plasma differentially regulates sperm–PMN binding and PMN activation. In Study 1, PMN activation was influenced by seminal plasma treatment, serum, and time, whereas binding was primarily treatment driven. The addition of serum, which introduces female-derived complement proteins, increased activation in SP-treated groups, but activation levels remained substantially lower than in FT controls, indicating that SP buffers complement-driven neutrophil responses. In serum-free conditions, whole SP and DSP suppressed PMN activation more rapidly than enriched EVs, supporting the concept that soluble proteins provide immediate immunomodulatory activity following semen deposition. Study 2 reinforced this pattern, as protective fractions F3 and F5 maintained suppression of PMN activation and binding even in the presence of serum. By contrast, EV-associated proteins are likely to provide a protected delivery platform that shields cargo from enzymatic degradation and enables sustained signaling beyond the regions of the female reproductive tract typically occupied by free seminal plasma (77). Together, these findings support complementary temporal roles for soluble and EV-associated proteins, with soluble factors rapidly buffering the initial inflammatory response and EVs providing prolonged regulation of sperm immune architecture.

Notably, patterns of PMN activation did not strictly mirror binding, which, when considered alongside the pro- and anti- inflammatory proteins within the core protective profile described above, supports that SP mediates a balance between activating and suppressive signals to orchestrate controlled immune engagement. Importantly, these effects occurred independently of sperm function, as motility, viability, and acrosome integrity were largely maintained, highlighting that neutrophil regulation is primarily mediated by extrinsic seminal plasma factors rather than selective targeting of poor-quality sperm. Collectively, these findings reinforce that seminal plasma regulates innate immunity through multiple complementary mechanisms involving both seminal plasma proteins and female-derived complement components.

While the mechanisms by which the female immune system recognizes spermatozoa remain incompletely defined, our findings identify complement as a key feature of sperm–PMN interactions. Classically, complement has been viewed as a female-driven cascade, with complement proteins and regulators present in female reproductive fluids and expressed by epithelial tissues of the FRT (25, 26). The present study, however, identifies a novel role for seminal plasma derived complement factors in modulating PMN responsiveness to ram spermatozoa (**Figure 5B**). Complement components were broadly distributed across seminal plasma fractions but exhibited fraction-dependent enrichment, suggesting functional compartmentalization rather than simple presence or absence. In particular, EV fractions were enriched in membrane-associated regulators, including CD46 and CD59, consistent with vesicle-mediated delivery to sperm surfaces, whereas soluble fractions were comparatively enriched in complement mediators and soluble regulators such as C3, C9, CFI, CFB, LTF, CLU, and CFH, suggesting complementary mechanisms of immune regulation. How these proteins are transferred to or expressed on spermatozoa remains unknown but may provide important insight into mechanisms of sperm immune evasion. Complement-associated proteins are conserved across domestic species (60, 61, 63), with lactoferrin (LTF), for example, shown in horses to selectively promote PMN binding to dead, but not viable, spermatozoa when complexed with superoxide dismutase (SOD-3) (78). This conservation supports a fundamental role for seminal plasma complement proteins in promoting sperm survival and immune tolerance within the female reproductive tract.

EVs were preferentially associated with CD46, a key anti-opsonin and CD59 an inhibitor of the membrane attack complex, which are intrinsically expressed on human spermatozoa (79). CD46 identified on bull spermatozoa has even been shown to be significantly diminished by cryopreservation (80, 81). Together, the presence of CD46 and CD59 in ram SPEVs, may facilitate vesicular delivery from seminal plasma to shield the sperm membrane which is then stripped or altered following *in vitro* processing. Its presence may, protect against phagocytosis and cell lysis by the complement system (79, 82, 83), when lost, this alters the immune response to sperm. Soluble complement inhibitory proteins, including Clusterin (CLU) and factor H (CFH) (84), appeared more dominant in DSP than EVs, suggesting they may be responsible for the observed “buffering” of complement-driven PMN activation observed *in vitro*. This dual-origin complement system illustrates how female serum/fluids and seminal plasma interact to define PMN activation and binding, balancing sperm protection with pathogen defense. Notably, many of these complement-associated proteins have not previously been characterized in ram seminal plasma or on the membrane of ram spermatozoa. Defining their localization and abundance across sperm maturation, ejaculation, and cryopreservation will be essential to determine whether complement regulators are developmentally acquired, selectively lost during freezing, and/or replenished via EV-mediated transfer post supplementation, providing a mechanistic theory to SP immune protection. Furthermore, functional assays to confirm the inhibitory activity of soluble SP-derived complement regulators will be critical to establish their mechanistic contribution to neutrophil modulation and sperm survival.

Pathway enrichment analyses further highlight divergent immunoregulatory mechanisms between EV- and soluble-associated proteins. Notably, many of the enriched pathways (including IL-8, CXCR4, fMLP, integrin, RAC/RHO GTPase and actin cytoskeleton signaling) regulate neutrophil chemotaxis, adhesion and activation, providing biological context for the observed differences in sperm–PMN interactions. Consistent with this, DSP proteins were enriched for predicted inhibition of innate immune pathways, including IL-8 signaling and neutrophil degranulation, whereas purified EVs were associated with predicted activation of regulatory and adaptive immune-related pathways, including Ephrin receptor and CXCR4 signaling. Taken together with our functional data, which showed that both DSP and EVs reduced PMN binding, these results suggest that while both fractions engage PMNs to modulate female immunity, the mechanisms by which they do so may differ. This potential mechanistic divergence may follow the temporal separation of soluble versus EV-associated proteins. However, these roles should not be considered strictly binary, as many candidate immunomodulatory proteins exist in both soluble and EV-associated forms, likely supporting robust, tiered immunoregulatory activity. This is exemplified by EDIL3, the most abundant EV cargo, which was also detected in soluble fractions, supporting multi-modal delivery to transcriptionally silent sperm and epithelial cells. EDIL3 has been suggested to potentially mask cryo-induced Damage-Associated Molecular Patterns (DAMPs) or modulate integrin-mediated adhesion (85, 86). In somatic systems, EDIL3 suppresses leukocyte recruitment and IL-17-driven inflammation (87–90), supporting its potential role in sperm protection. Its high abundance in ram SP-EVs, coupled with its previously identified contribution to the ejaculated sperm proteome (85), identifies EDIL3 as an additional candidate for future studies investigating EV-mediated immune protection of spermatozoa.

Reliable isolation and validation of SP-EVs are essential for interpreting downstream functional and pathway enrichment analyses. The mechanistic findings of this study are supported by comprehensive physical and proteomic characterization of ram SP-EVs, providing a robust platform to investigate reproductive immune interactions in livestock. EVs were isolated from pooled seminal plasma collected from 26 rams across multiple ejaculates, minimizing inter-individual variation and enabling identification of conserved vesicle-associated proteins independent of male-specific effects. In accordance with MISEV2023 guidelines (49), the preparations were enriched for canonical EV markers, effectively separated from soluble seminal plasma proteins, and recovered at physiologically relevant concentrations comparable to those reported in humans (45). SP-EVs have also been described in bulls, boars and stallions (91–94), suggesting evolutionary conservation of vesicle-mediated signaling, although comparative analyses of EV cargo and function across species remain limited. Future studies examining ejaculate-specific cargo variation and ram-to-ram heterogeneity will be important for defining individual contributions to reproductive immune modulation and fertility outcomes.

Beyond direct immunomodulation and PMN interactions, seminal plasma contributes to maintenance of the sperm molecular coating, including glycocalyx-associated proteins that regulate cellular interactions and mask paternal antigens. CRISPs 1–3 and β-defensins (e.g., DEFB124) were identified within protective fractions in the present study and are known components of the sperm glycocalyx, potentially contributing to immune invisibility within the female reproductive tract. Ovine CRISP-1, an epididymal-derived acidic glycoprotein, has a predicted molecular weight consistent with potential enrichment in the 30–50 kDa fraction (F3) (95), although further characterization of ovine CRISPs is required given DDA-based molecular weight estimates for CRISP2 and CRISP3 (~27 kDa). CRISP isoforms exhibit broad conservation across mammals, with CRISP1–3 reported in human, stallion, and boar seminal plasma, CRISP1–2/4 additionally described in rodents, and species-specific expression patterns suggesting divergent roles across reproductive systems (60, 96, 97). Notably, CRISP1, CRISP2 and CRISP3 have previously been identified in ram seminal plasma (60), while their presence in ram SP-EVs is reported here for the first time. Although traditionally studied for sperm–egg interactions, evidence from stallion spermatozoa, where CRISP3 contributes to protection against PMN binding (30, 98), together with the current findings, suggests CRISPs may also contribute to immune regulation and sperm transport within the female reproductive tract.

We identified β-defensins DEFB119, DEFB123, and DEFB129 across SP, DSP, EV, and F5, while DEFB124 was detected in all protective fractions. Given the conserved structural features shared among β-defensins, it is plausible that DEFB124 may contribute to immunomodulatory functions analogous to those described for DEFB126 at the sperm surface, including masking of sperm antigens and participation in glycan-mediated immune signaling (99). In particular, sperm glycans, especially sialic acids and glycosaminoglycans, can function as self-associated molecular patterns (SAMPs) that engage inhibitory Siglec receptors on epithelial and immune cells within the female reproductive tract, thereby promoting immune quiescence (100–103). Importantly, glycan-mediated immune signaling and complement regulation are likely interconnected. Sialylated sperm glycans can recruit complement regulatory proteins such as factor H while simultaneously engaging inhibitory Siglec receptors on leukocytes, providing a dual mechanism for limiting innate immune recognition. Although the extensive glycosylation characteristic of DEFB126 has not yet been demonstrated for DEFB124, β-defensins can associate with sperm membranes and glycocalyx components, raising the possibility that DEFB124 contributes to shaping the sperm surface environment and modulating immune recognition.

Together, these proteins highlight the importance of maintaining the molecular coating of spermatozoa, which may underpin their unique immune privilege within the female reproductive tract. Cryopreservation is known to disrupt the molecular identity of spermatozoa, and we hypothesize that this process compromises glycocalyx integrity, exposing previously masked sperm antigens. Such disruption may impair the ability of the female immune system to recognize sperm as tolerable cells, instead promoting indiscriminate phagocytic clearance by polymorphonuclear leukocytes. Characterizing the sperm glycocalyx and its associated seminal plasma components therefore represents an important additional layer of sperm molecular identity, which must be considered alongside the broader contributions of SP itself, to fully understand how immune tolerance to sperm is established, maintained, and potentially restored. This perspective highlights that immune privilege is unlikely to rely on a single protein candidate, but rather arises from a coordinated, multi-layered system of molecular and vesicle-associated factors.

In summary, this study demonstrates that seminal plasma and its interactions with spermatozoa function as a structured immunoregulatory system rather than a passive reproductive fluid. Distinct soluble and vesicular compartments cooperate to modulate neutrophil engagement, buffering complement-driven activation while maintaining controlled innate immune responses within the female reproductive tract. Extracellular vesicles provide a protected platform for delivery of immune-regulatory cargo, while soluble proteins facilitate rapid local modulation following semen deposition. Together with glycocalyx-associated proteins, these mechanisms may contribute to sperm immune privilege by maintaining an appropriate balance between immune recognition and tolerance. The identification of a conserved seminal plasma protein signature associated with reduced sperm–PMN binding provides a foundation for defining the molecular determinants of reproductive immune regulation and successful sperm transport. Future studies should investigate sperm–EV interactions, validate candidate immunomodulators on different sperm populations, and determine how cryopreservation disrupts this protective signaling network. By defining the molecular architecture through which seminal plasma regulates neutrophil behavior, these findings position seminal plasma extracellular vesicles as important mediators of sperm immune tolerance and highlight opportunities to improve fertility outcomes and assisted reproductive technologies.

## Data Availability

The original contributions presented in the study are publicly available. This data can be found here.

## References

[1] Warr S Pini T De Graaf SP Rickard JP . Molecular insights to the sperm–cervix interaction and the consequences for cryopreserved sperm. Biol Reprod. (2023) 108:183–96. doi: 10.1007/978-3-319-47446-5_8 36191077

[2] Salamon S Maxwell WMC . Storage of ram semen. Anim Reprod Sci. (2000) 62:77–111. doi: 10.1016/s0378-4320(00)00155-x 10924821

[3] Lightfoot RJ Salamon S . Fertility of ram spermatozoa frozen by the pellet method. J Reprod Fertility. (1970) 22:385–98. doi: 10.1530/jrf.0.0220399 5453373

[4] Maxwell W Hewitt L . A comparison of vaginal, cervical and intrauterine insemination of sheep. J Agric Sci. (1986) 106:191–3. doi: 10.1017/s0021859600061906 41292463

[5] Gillan L Maxwell WM . The functional integrity and fate of cryopreserved ram spermatozoa in the female tract. J Reprod Fertil Suppl. (1999) 54:271–83. doi: 10.1530/biosciprocs.4.021 10692861

[6] Pini T Leahy T de Graaf SP . Sublethal sperm freezing damage: Manifestations and solutions. Theriogenology. (2018) 181:172–81. 29913422 10.1016/j.theriogenology.2018.06.006

[7] Dohadwala S Shah P Farrell MK Politch JA Marathe J Costello CE . Sialidases derived from Gardnerella vaginalis and Prevotella timonensis remodel the sperm glycocalyx and impair sperm function. Glycobiology. (2025) 35(11):cwaf067. doi: 10.1093/glycob/cwaf067 41165775 PMC12599315

[8] Maxwell W Evans G Mortimer S Gillan L Gellatly E Mcphie C . Normal fertility in ewes after cervical insemination with frozen–thawed spermatozoa supplemented with seminal plasma. Reprod Fertil Dev. (1999) 11:123–6. doi: 10.1071/rd99046 10735556

[9] Rickard J Pini T Soleilhavoup C Cognié J Bathgate R Lynch G . Seminal plasma aids the survival and cervical transit of epididymal ram spermatozoa. Reproduction. (2014) 148:469–78. doi: 10.1530/rep-14-0285e 25118301

[10] Chen G Nunez G . Sterile inflammation: sensing and reacting to damage. Nat Rev Immunol. (2010) 10:826–37. doi: 10.1038/nri2873 21088683 PMC3114424

[11] Schaefer TM Desouza K Fahey JV Beagley KW Wira CR . Toll‐like receptor (TLR) expression and TLR‐mediated cytokine/chemokine production by human uterine epithelial cells. Immunology. (2004) 112:428–36. doi: 10.1111/j.1365-2567.2004.01898.x 15196211 PMC1782499

[12] Katila T . Post‐mating inflammatory responses of the uterus. Reprod Domest Anim. (2012) 47:31–41. doi: 10.1111/j.1439-0531.2012.02120.x 22913558

[13] Matthijs A Engel B Woelders H . Neutrophil recruitment and phagocytosis of boar spermatozoa after artificial insemination of sows, and the effects of inseminate volume, sperm dose and specific additives in the extender. Reproduction. (2003) 125:357–67. doi: 10.1530/rep.0.1250357 12611599

[14] Scott JL Ketheesan N Summers PM . Leucocyte population changes in the reproductive tract of the ewe in response to insemination. Reprod Fertil Dev. (2006) 18:627–34. doi: 10.1071/rd05165 16930509

[15] Sharkey DJ Tremellen KP Jasper MJ Gemzell-Danielsson K Robertson SA . Seminal fluid induces leukocyte recruitment and cytokine and chemokine mRNA expression in the human cervix after coitus. J Immunol. (2012) 188:2445–54. doi: 10.4049/jimmunol.1102736 22271649

[16] Schjenken JE Robertson SA . The female response to seminal fluid. Physiol Rev. (2020) 100(3):1077–17. doi: 10.1152/physrev.00013.2018 31999507

[17] Thompson L Barratt C Bolton A Cooke I . The leukocytic reaction of the human uterine cervix. Am J Reprod Immunol. (1992) 28:85–9. doi: 10.1111/j.1600-0897.1992.tb00765.x 1285856

[18] De M Choudhuri R Wood GW . Determination of the number and distribution of macrophages, lymphocytes, and granulocytes in the mouse uterus from mating through implantation. J Leukocyte Biol. (1991) 50:252–62. doi: 10.1002/jlb.50.3.252 1856596

[19] Howe G Black D . Spermatozoan transport and leucocytic responses in the reproductive tract of calves. Reproduction. (1963) 6:305–11. doi: 10.1530/jrf.0.0060305 14073737

[20] Mattner P . Differential leucocytic responses to spermatozoa in the cervix and the uterus in ewes. Reproduction. (1969) 18:297–303. doi: 10.1530/jrf.0.0180297 5815308

[21] Rozeboom K Troedsson M Crabo B . Characterization of uterine leukocyte infiltration in gilts after artificial insemination. Reproduction. (1998) 114:195–9. doi: 10.1530/jrf.0.1140195 10070347

[22] Katila T . Onset and duration of uterine inflammatory response of mares after insemination with fresh semen. Biol Reprod. (1995) 52:515–7. doi: 10.1093/biolreprod/52.monograph_series1.515 40388063

[23] Scott JL Ketheesan N Summers PM . Spermatozoa and seminal plasma induce a greater inflammatory response in the ovine uterus at oestrus than dioestrus. Reprod Fertil Dev. (2009) 21(7):817–26. doi: 10.1071/RD09012 19698286

[24] Rosales C . Neutrophil: a cell with many roles in inflammation or several cell types? Front Physiol. (2018) 9:113. doi: 10.3389/fphys.2018.00113 29515456 PMC5826082

[25] Vanderpuye OA Labarrere CA Mcintyre JA . The complement system in human reproduction. Am J Reprod Immunol. (1992) 27:145–55. doi: 10.1111/j.1600-0897.1992.tb00742.x 1384536

[26] Oglesby TJ Longwith JE Huettner PC . Human complement regulator expression by the normal female reproductive tract. Anat Rec. (1996) 246:78–86. doi: 10.1002/(sici)1097-0185(199609)246:1<78::aid-ar9>3.0.co;2-b 8876826

[27] Janewa CA Jr Medzhitov R . Innate immune recognition. Annu Rev Immunol. (2002) 20(1):197–216. doi: 10.1146/annurev.immunol.20.083001.084359 11861602

[28] Alghamdi AS Lovaas BJ Bird SL Lamb GC Rendahl AK Taube PC . Species-specific interaction of seminal plasma on sperm-neutrophil binding. Anim Reprod Sci. (2009) 114(4):331–44. doi: 10.1016/j.anireprosci.2008.10.015 19081210

[29] Donnellan E O'Brien M Meade KG Fair S . Comparison of the uterine inflammatory response to frozen-thawed sperm from high and low fertility bulls. Theriogenology. (2021) 176:26–34. doi: 10.1016/j.theriogenology.2021.09.012 34564014

[30] Doty A Buhi WC Benson S Scoggin KE Pozor M Macpherson M . Equine CRISP3 modulates interaction between spermatozoa and polymorphonuclear neutrophils1. Biol Reprod. (2011) 85:157–64. doi: 10.1095/biolreprod.110.084491 21389342

[31] Donnellan EM Cormican P Reid C Duggan G Stiavnicka M Meade KG . The transcriptomic response of bovine uterine tissue is altered in response to sperm from high-and low-fertility bulls. Biol Reprod. (2023) 108(6):912–21. doi: 10.1093/biolre/ioad031 PMC10266941 36947086

[32] Zambrano F Namuncura C Uribe P Schulz M Pezo F Burgos R . Swine spermatozoa trigger aggregated neutrophil extracellular traps leading to adverse effects on sperm function. J Reprod Immunol. (2021) 146:103339. doi: 10.1016/j.jri.2021.103339 34087539

[33] Zambrano F Pezo F Furugen Cesar De Andrade A Rivera-Concha R Uribe P Schulz M . Modulation of NETosis in swine neutrophil–spermatozoa co-cultures *in vitro*: Effects of butylated hydroxytoluene, albumin, prostaglandin E2, and seminal plasma. Antioxidants. (2025) 14:778. doi: 10.3390/antiox14070778 40722882 PMC12291730

[34] Mateo-Otero Y Zambrano F Catalán J Sánchez R Yeste M Miro J . Seminal plasma, and not sperm, induces time and concentration-dependent neutrophil extracellular trap release in donkeys. Equine Vet J. (2022) 54(2):415–26. doi: 10.1111/evj.13457 33908643

[35] Zambrano F Carrau T Gärtner U Seipp A Taubert A Felmer R . Leukocytes coincubated with human sperm trigger classic neutrophil extracellular traps formation, reducing sperm motility. Fertil Steril. (2016) 106(5):1053–60.e1051. doi: 10.1016/j.fertnstert.2016.06.005 27344301

[36] Mikhael L Warr S De Graaf SP Rickard JP . Evaluating sperm–polymorphonuclear leukocyte interactions *in vitro*: the immunomodulatory role of seminal plasma and implications for future ovine studies. Reprod Fertil Dev. (2026) 38:RD25131. doi: 10.1071/rd25131 41385721

[37] Pini T Leahy T Paul De Graaf S . Seminal plasma and cryopreservation alter ram sperm surface carbohydrates and interactions with neutrophils. Reprod Fertil Dev. (2018) 30:689–702. doi: 10.1071/rd17251 29065974

[38] Warr S Pini T De Graaf SP Rickard JP . Seminal plasma protects frozen-thawed ram spermatozoa from neutrophil attack: a complement mediated dynamic. Reproduction. (2025) 170(2). doi: 10.1530/rep-24-0465 40536802 PMC12231180

[39] Alghamdi AS Foster DN Troedsson MHT . Equine seminal plasma reduces sperm binding to polymorphonuclear neutrophils (PMNs) and improves the fertility of fresh semen inseminated into inflamed uteri. Reproduction. (2004) 127:593–600. doi: 10.1530/rep.1.00096 15129015

[40] Troedsson M Desvousges A Alghamdi A Dahms B Dow C Hayna J . Components in seminal plasma regulating sperm transport and elimination. Anim Reprod Sci. (2005) 89:171–86. doi: 10.1016/j.anireprosci.2005.07.005 16102920

[41] Li J-C Yamaguchi S Funahashi H . Boar seminal plasma or hen's egg yolk decrease the in-vitro chemotactic and phagocytotic activities of neutrophils when co-incubated with boar or bull sperm. Theriogenology. (2012) 77:73–80. doi: 10.1016/j.theriogenology.2011.07.018 21872309

[42] Gadella B . Sperm surface proteomics. In: Immune Infertility: The Impact of Immune Reactions on Human Infertility. Berlin, Heidelberg: Springer Berlin Heidelberg. (2009). p. 33–48.

[43] Leahy T Rickard JP Bernecic NC Druart X de Graaf SP . Ram seminal plasma and its functional proteomic assessment. Reproduction. (2019) 157(6):R243–R256. doi: 10.1530/REP-18-0627 30844754

[44] Leahy T Rickard JP Pini T Gadella BM De Graaf SP . Quantitative proteomic analysis of seminal plasma, sperm membrane proteins, and seminal extracellular vesicles suggests vesicular mechanisms aid in the removal and addition of proteins to the ram sperm membrane. Proteomics. (2020) 20:1900289. doi: 10.1002/pmic.201900289 32383290

[45] Tamessar CT Anderson AL Bromfield EG Trigg NA Parameswaran S Stanger SJ . The efficacy and functional consequences of interactions between human spermatozoa and seminal fluid extracellular vesicles. Reprod Fertility. (2024) 5(4). doi: 10.1530/raf-23-0088 39230058 PMC11466262

[46] Torres-Arce E Tamessar CT Robertson SA Nixon B Sharkey DJ Schjenken JE . Exploring the contributions of human seminal extracellular vesicles to reproduction and fertility. Reproduction. (2026) 171(1). doi: 10.1093/reprod/xaaf006 41582538

[47] Ronquist G . Prostasomes: Their Characterisation: Implications for Human Reproduction: Prostasomes and Human Reproduction. Adv Exp Med Biol. (2015) 868:191–209. doi: 10.1007/978-3-319-18881-2_9 PMC7120776 26178851

[48] Milutinović B Goč S Mitić N Kosanović M Janković M . Surface glycans contribute to differences between seminal prostasomes from normozoospermic and oligozoospermic men. Upsala J Med Sci. (2019) 124(2):111–8. doi: 10.1080/03009734.2019.1592266 PMC6566730 30957617

[49] Welsh JA Goberdhan DC O'Driscoll L Buzas EI Blenkiron C Bussolati B . Minimal information for studies of extracellular vesicles (MISEV2023): From basic to advanced approaches. J Extracell Vesicles. (2024) 13(2):e12404. doi: 10.1002/jev2.12404 38326288 PMC10850029

[50] Brzozowski J Bond D Jankowski H Goldie B Burchell R Naudin C . Extracellular vesicles with altered tetraspanin CD9 and CD151 levels confer increased prostate cell motility and invasion. Sci Rep. (2018) 8:8822. doi: 10.1038/s41598-018-27180-z 29891991 PMC5995928

[51] Miró J Catalán J Marín H Yánez-Ortiz I Yeste M . Specific seminal plasma fractions are responsible for the modulation of sperm–PMN binding in the donkey. Animals. (2021) 11:1388. doi: 10.3390/ani11051388 34068214 PMC8153123

[52] Evans G Maxwell WC . Salamons' Artificial Insemination of Sheep and Goats. Sydney: Butterworths (1987).

[53] Spanner EA Rickard JP De Graaf SP . The effect of freezing and thawing techniques on the *in vitro* quality of pellet-frozen ram semen. Anim Reprod Sci. (2025) 276:107822. doi: 10.1016/j.anireprosci.2025.107822 40233545

[54] Sherman BT Hao M Qiu J Jiao X Baseler MW Lane HC . DAVID: a web server for functional enrichment analysis and functional annotation of gene lists, (2021 update). Nucleic Acids Res. (2022) 50:W216–21. doi: 10.1093/nar/gkac194 35325185 PMC9252805

[55] Huang Da W Sherman BT Lempicki RA . Systematic and integrative analysis of large gene lists using DAVID bioinformatics resources. Nat Protoc. (2009) 4:44–57. doi: 10.1038/nprot.2008.211 19131956

[56] Pini T De Graaf SP Druart X Tsikis G Labas V Teixeira-Gomes AP . Binder of Sperm Proteins 1 and 5 have contrasting effects on the capacitation of ram spermatozoa. Biol Reprod. (2018) 98(6):765–75. 10.1093/biolre/ioy032 29415221

[57] Alghamdi AS Foster DN . Seminal DNase frees spermatozoa entangled in neutrophil extracellular traps. Biol Reprod. (2005) 73(6):1174–81. doi: 10.1095/biolreprod.105.045666 16107606

[58] Alghamdi AS Lovaas BJ Bird SL Lamb GC Rendahl AK Taube PC . Species-specific interaction of seminal plasma on sperm-neutrophil binding. Anim Reprod Sci. (2009) 114(4):331–44. doi: 10.1016/j.anireprosci.2008.10.015 19081210

[59] Doty AL Miller LM Fedorka CE Troedsson MH . The role of equine seminal plasma derived cysteine rich secretory protein 3 (CRISP3) in the interaction between polymorphonuclear neutrophils (PMNs) and populations of viable or dead spermatozoa, and bacteria. Theriogenology. (2024) 219:22–31. doi: 10.1016/j.theriogenology.2024.02.014 38377715

[60] Soleilhavoup C Tsikis G Labas V Harichaux G Kohnke P Dacheux J-L . Ram seminal plasma proteome and its impact on liquid preservation of spermatozoa. J Proteomics. (2014) 109:245–60. doi: 10.1016/j.jprot.2014.07.007 25053255

[61] Druart X Rickard J Mactier S Kohnke P Kershaw-Young C Bathgate R . Proteomic characterization and cross species comparison of mammalian seminal plasma. Proteomics. (2013) 91:13–22. doi: 10.1016/j.jprot.2013.05.029 23748023

[62] Menezes EB De Oliveira RV Van Tilburg MF Barbosa EA Nascimento NV Velho ALMCS . Proteomic analysis of seminal plasma from locally-adapted “Curraleiro Pé-Duro bulls” (Bos taurus): identifying biomarkers involved in sperm physiology in endangered animals for conservation of biodiversity. Anim Reprod Sci. (2017) 183:86–101. doi: 10.1016/j.anireprosci.2017.05.014 28625714

[63] Perez-Patino C Barranco I Parrilla I Valero ML Martinez EA Rodriguez-Martinez H . Characterization of the porcine seminal plasma proteome comparing ejaculate portions. J Proteomics. (2016) 142:15–23. doi: 10.1016/j.jprot.2016.04.026 27109353

[64] Pini T Nixon B Karr TL Teperino R Sanz-Moreno A Da Silva-Buttkus P . Towards a kingdom of reproductive life - the core sperm proteome. Reproduction. (2025) 169:e250105. doi: 10.1530/rep-25-0105 40298292 PMC12070448

[65] Wang X Cui X Zhu C Li M Zhao J Shen Z . FKBP11 protects intestinal epithelial cells against inflammation-induced apoptosis via the JNK-caspase pathway in Crohn's disease. Mol Med Rep. (2018) 18(5):4428–38. doi: 10.3892/mmr.2018.9485 PMC6172375 30221722

[66] Boligala GP Yang MV van Wunnik JC Pruitt K . Nuclear Dishevelled: An enigmatic role in governing cell fate and Wnt signaling. Biochim Biophys Acta Mol Cell Res. (2022) 1869(10):119305. doi: 10.1016/j.bbamcr.2022.119305 35688346

[67] Castro-Piedras I Sharma M Brelsfoard J Vartak D Martinez EG Rivera C . Nuclear Dishevelled targets gene regulatory regions and promotes tumor growth. EMBO Rep. (2021) 22(6):e50600. doi: 10.15252/embr.202050600 33860601 PMC8183407

[68] Peer D Park EJ Morishita Y Carman CV Shimaoka M . Systemic leukocyte-directed siRNA delivery revealing Cyclin D1 as an anti-inflammatory target. Science. (2008) 319(5863):627–30. doi: 10.1126/science.1149859 PMC2490797 18239128

[69] Jang G-Y Kim YS Lee SE Lee JW Han HD Kang TH . Improvement of DC-based vaccines using adjuvant TLR4-binding 60S acidic ribosomal protein P2 and immune checkpoint inhibitors. Cancer Immunol Immunother. (2021) 70:1075–88. doi: 10.1007/s00262-020-02759-6 33113002 PMC10992392

[70] Schjenken JE Glynn DJ Sharkey DJ Robertson SA . TLR4 signaling is a major mediator of the female tract response to seminal fluid in mice1. Biol Reprod. (2015) 93(3):68–1. doi: 10.1095/biolreprod.114.125740 26157066

[71] Li M Mennone A Soroka CJ Hagey LR Ouyang X Weinman EJ . Na^+^/H^+^ exchanger regulatory factor 1 knockout mice have an attenuated hepatic inflammatory response and are protected from cholestatic liver injury. Hepatology. (2015) 62(4):1227–36. doi: 10.1002/hep.27956 PMC4589453 26108984

[72] Kammala AK Sheller-Miller S Radnaa E Kechichian T Subramanian H Menon R . Sodium hydrogen exchanger regulatory factor-1 (NHERF1) regulates fetal membrane inflammation. Int J Mol Sci. (2020) 21(20):7747. doi: 10.3390/ijms21207747 33092043 PMC7589612

[73] Ma R Tang Z Wang J . PTTG1IP (PBF) is a prognostic marker and correlates with immune infiltrate in ovarian cancer. Am J Transl Res. (2023) 15(1):27. PMC9908464 36777854

[74] Thomas GMH Cunningham E Fensome A Ball A Totty NF Truong O . An essential role for phosphatidylinositol transfer protein in phospholipase C-mediated inositol lipid signaling. Cell. (1993) 74:919–28. doi: 10.1016/0092-8674(93)90471-2 8374957

[75] Jakus Z Simon E Frommhold D Sperandio M Mócsai A . Critical role of phospholipase Cγ2 in integrin and Fc receptor-mediated neutrophil functions and the effector phase of autoimmune arthritis. J Exp Med. (2009) 206:577–93. doi: 10.1083/jcb1846oia15 PMC2699137 19273622

[76] Kular G Loubtchenkov M Swigart P Whatmore J Ball A Cockcroft S . Co-operation of phosphatidylinositol transfer protein with phosphoinositide 3-kinase γ in the formylmethionyl-leucylphenylalanine-dependent production of phosphatidylinositol 3,4,5-trisphosphate in human neutrophils. Biochem J. (1997) 325:299–301. doi: 10.1042/bj3250299 9230105 PMC1218559

[77] Suarez SS Pacey AA . Sperm transport in the female reproductive tract. Hum Reprod Update. (2005) 12:23–37. doi: 10.1093/humupd/dmi047 16272225

[78] Alghamdi AS Fedorka CE Scoggin KE Esteller-Vico A Beatty K Davolli G . Binding of equine seminal lactoferrin/superoxide dismutase (SOD-3) complex is biased towards dead spermatozoa. Animals. (2022) 13:52. doi: 10.3390/ani13010052 36611662 PMC9817809

[79] Simpson K Holmes C . Differential expression of complement regulatory proteins decay-accelerating factor (CD55), membrane cofactor protein (CD46) and CD59 during human spermatogenesis. Immunology. (1994) 81:452. 7515850 PMC1422347

[80] Jankovicova J Simon M Antalíková J Horovská Ľ Fábryová K . Potential role of bovine CD46 in the reproduction process. J Agrobiology. (2008) 25:35–7.

[81] Jankovicova J Antalikova J Simon M Michalkova K Horovska L . Comparative fluorescence analysis of the bovine sperm using IVA^-5^20 (anti-CD46 antibody) and lectins: probable localisation of CD46 on bovine sperm membrane. Gen Physiol Biophys. (2011) 30:70–6. doi: 10.4149/gpb_2011_si1_70 21869454

[82] Sakaue T Takeuchi K Maeda T Yamamoto Y Nishi K Ohkubo I . Factor H in porcine seminal plasma protects sperm against complement attack in genital tracts. J Biol Chem. (2010) 285(3):2184–92. doi: 10.1074/jbc.M109.063495 PMC2804374 19920146

[83] Tschopp J Chonn A Hertig S French LE . Clusterin, the human apolipoprotein and complement inhibitor, binds to complement C7, C8 beta, and the b domain of C9. J Immunol. (1993) 151(4):2159–65. doi: 10.4049/jimmunol.151.4.2159 8345200

[84] Sarma JV Ward PA . The complement system. Cell Tissue Res. (2011) 343:227–35. doi: 10.1007/s00441-010-1034-0 20838815 PMC3097465

[85] Pini T Leahy T Soleilhavoup C Tsikis G Labas V Combes-Soia L . Proteomic investigation of ram spermatozoa and the proteins conferred by seminal plasma. J Proteome Res. (2016) 15:3700–11. doi: 10.1021/acs.jproteome.6b00530 27636150

[86] Gao C Li J Feng F Fu J Gong T Cao S . EDIL3 regulates pulmonary artery smooth muscle cell proliferation and migration via integrin αVβ3/ERK1/2 axis in pulmonary hypertension. Eur J Pharmacol. (2025):177901. doi: 10.1016/j.ejphar.2025.177901 40609616

[87] Choi EY Chavakis E Czabanka MA Langer HF Fraemohs L Economopoulou M . Del-1, an endogenous leukocyte-endothelial adhesion inhibitor, limits inflammatory cell recruitment. Science. (2008) 322(5904):1101–4. doi: 10.1126/science.1165218 PMC2753175 19008446

[88] Eskan MA Jotwani R Abe T Chmelar J Lim J-H Liang S . The leukocyte integrin antagonist Del-1 inhibits IL-17-mediated inflammatory bone loss. Nat Immunol. (2012) 13(5):465–73. doi: 10.1038/ni.2260 PMC3330141 22447028

[89] Lee SJ Lee J Kim WW Jung JH Park HY Park J-Y . Del-1 expression as a potential biomarker in triple-negative early breast cancer. Oncology. (2018) 94(4):243–56. doi: 10.1159/000485658 29393238

[90] Kun Z Xin G Tao W Chenglong Z Dongsheng W Liang T . Tumor derived EDIL3 modulates the expansion and osteoclastogenesis of myeloid derived suppressor cells in murine breast cancer model. J Bone Oncol. (2019) 16:100238. doi: 10.1016/j.jbo.2019.100238 31110935 PMC6512748

[91] Carossino M Dini P Kalbfleisch TS Loynachan AT Canisso IF Shuck KM . Downregulation of microRNA eca-mir-128 in seminal exosomes and enhanced expression of CXCL16 in the stallion reproductive tract are associated with long-term persistence of equine arteritis virus. J Virol. (2018) 92(9):e00015-18. doi: 10.1128/JVI.00015-18 29444949 PMC5899189

[92] Xu Z Xie Y Wu C Gu T Zhang X Yang J . The effects of boar seminal plasma extracellular vesicles on sperm fertility. Theriogenology. (2024) 213:79–89. doi: 10.1016/j.theriogenology.2023.09.026 37816296

[93] Xu Z Xie Y Zhou C Hu Q Gu T Yang J . Expression pattern of seminal plasma extracellular vesicle small RNAs in boar semen. Front Vet Sci. (2020) 7:585276. doi: 10.3389/fvets.2020.585276 33263017 PMC7685987

[94] Shamsi RR Jozani RJ Asadpour R Rahbar M Taravat M . Seminal plasma-derived exosome preserves the quality parameters of the post-thaw semen of bulls with low freezeability. Biopreserv Biobank. (2025) 23(4):364–73. doi: 10.1089/bio.2024.0077 39723439

[95] Jorasia K Paul RK Rathore NS Lal P Singh R Sareen M . Production of bioactive recombinant ovine cysteine-rich secretory protein 1 in Escherichia coli. Syst Biol Reprod Med. (2021) 67:471–81. doi: 10.1080/19396368.2021.1963012 34459353

[96] Koppers AJ Reddy T O'Bryan MK . The role of cysteine-rich secretory proteins in male fertility. Asian J Androl. (2010) 13(1):111. doi: 10.1038/aja.2010.77 PMC3739402 20972450

[97] Töpfer-Petersen E Romero A Varela PF . Spermadhesins: a new protein family. Facts, hypotheses and perspectives. Andrologia. (1998) 30(4-5):217–24. doi: 10.1111/j.1439-0272.1998.tb01163.x 9739418

[98] Magdaleno L Gasset MA Varea J Schambony AM Urabanke C Raida M . Biochemical and conformational characterisation of HSP-3, a stallion seminal plasma protein of the cysteine-rich secretory protein (CRISP) family. FEBS Lett. (1997) 420:179–85. doi: 10.1016/s0014-5793(97)01514-7 9459306

[99] Tecle E Gagneux P . Sugar-coated sperm: Unraveling the functions of the mammalian sperm glycocalyx. Mol Reprod Dev. (2015) 82(9):635–50. doi: 10.1002/mrd.22500 PMC4744710 26061344

[100] Toshimori K Araki S Öra C Eddy E . Loss of sperm surface sialic acid induces phagocytosis: an assay with a monoclonal antibody T21, which recognizes a 54K sialoglycoprotein. Arch Androl. (1991) 27(2):79–86. doi: 10.3109/01485019108987656 1953200

[101] Koyama K Hasegawa A Komori S . Functional aspects of CD52 in reproduction. J Reprod Immunol. (2009) 83(1-2):56–9. doi: 10.1016/j.jri.2009.06.263 19875176

[102] Varki A . Letter to the glyco-forum: since there are PAMPs and DAMPs, there must be SAMPs? Glycan “self-associated molecular patterns” dampen innate immunity, but pathogens can mimic them. Glycobiology. (2011) 21(9):1121–24. doi: 10.1093/glycob/cwr087 PMC3150115 21932452

[103] Tecle E Gagneux P . Sugar-coated sperm: Unraveling the functions of the mammalian sperm glycocalyx. Mol Reprod Dev. (2015) 82(9):635–50. doi: 10.1002/mrd.22500 PMC4744710 26061344

